# Macrophage-derived PDGF-B induces muscularization in murine and human pulmonary hypertension

**DOI:** 10.1172/jci.insight.139067

**Published:** 2021-03-22

**Authors:** Aglaia Ntokou, Jui M. Dave, Amy C. Kauffman, Maor Sauler, Changwan Ryu, John Hwa, Erica L. Herzog, Inderjit Singh, W. Mark Saltzman, Daniel M. Greif

**Affiliations:** 1 Yale Cardiovascular Research Center, Section of Cardiovascular Medicine,; 2 Department of Genetics,; 3 Department of Biomedical Engineering,; 4 Section of Pulmonary, Critical Care and Sleep Medicine, Department of Internal Medicine, and; 5 Department of Pathology, Yale University, New Haven, Connecticut, USA.

**Keywords:** Pulmonology, Vascular Biology, Cardiovascular disease, Hypoxia, Macrophages

## Abstract

Excess macrophages and smooth muscle cells (SMCs) characterize many cardiovascular diseases, but crosstalk between these cell types is poorly defined. Pulmonary hypertension (PH) is a lethal disease in which lung arteriole SMCs proliferate and migrate, coating the normally unmuscularized distal arteriole. We hypothesized that increased macrophage platelet-derived growth factor–B (PDGF-B) induces pathological SMC burden in PH. Our results indicate that clodronate attenuates hypoxia-induced macrophage accumulation, distal muscularization, PH, and right ventricle hypertrophy (RVH). With hypoxia exposure, macrophage *Pdgfb* mRNA was upregulated in mice, and *LysM‑Cre* mice carrying floxed alleles for *hypoxia-inducible factor 1a*, *hypoxia-inducible factor 2a*, or *Pdgfb* had reduced macrophage Pdgfb and were protected against distal muscularization and PH. Conversely, *LysM‑Cre von-Hippel Lindau^fl/fl^* mice had increased macrophage Hifa and Pdgfb and developed distal muscularization, PH, and RVH in normoxia. Similarly, Pdgfb was upregulated in macrophages from human idiopathic or systemic sclerosis–induced pulmonary arterial hypertension patients, and macrophage-conditioned medium from these patients increased SMC proliferation and migration via PDGF-B. Finally, in mice, orotracheal administration of nanoparticles loaded with Pdgfb siRNA specifically reduced lung macrophage Pdgfb and prevented hypoxia-induced distal muscularization, PH, and RVH. Thus, macrophage-derived PDGF-B is critical for pathological SMC expansion in PH, and nanoparticle-mediated inhibition of lung macrophage PDGF-B has profound implications as an interventional strategy for PH.

## Graphical abstract

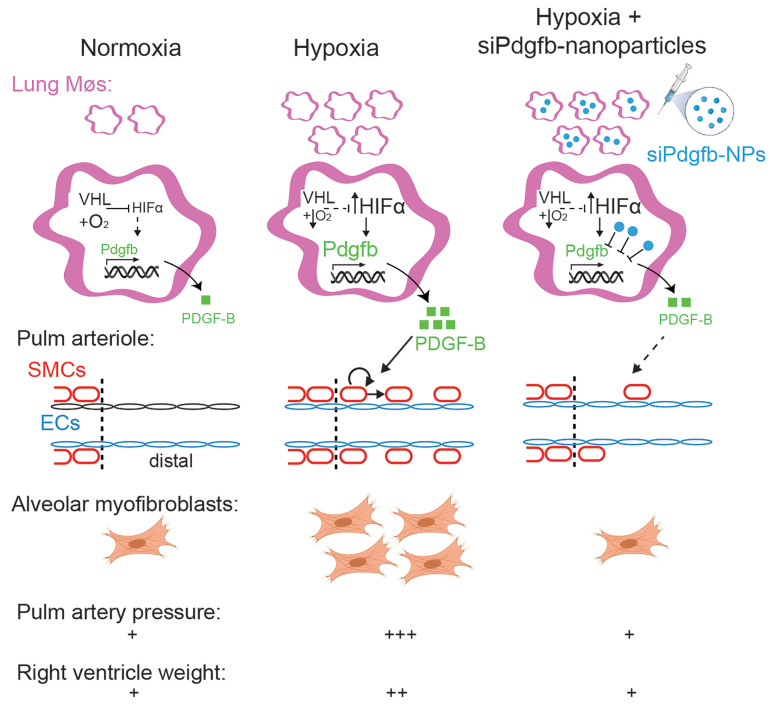

## Introduction

Cardiovascular diseases, such as pulmonary hypertension (PH), have a major deleterious impact on human health. Indeed, PH, which is defined by a mean pulmonary arterial pressure greater than 20 mmHg, is responsible for more than 20,000 deaths annually in the United States alone (1, 2). PH is composed of a heterogenous collection of clinical conditions that are classified into 5 groups by the World Health Organization (WHO) based on clinical presentation, hemodynamics, pathological findings, and therapies (1). Herein, we focus on WHO Group 1, or pulmonary arterial hypertension (PAH), which includes idiopathic (IPAH; formerly classified as primary PH), and Group 3, which is due to lung diseases and/or hypoxia. Approximately one-half of PAH cases are IPAH, heritable, or drug induced, and another important subgroup are associated PAH conditions of which the leading cause is connective tissue disease, predominantly systemic sclerosis (SSc; also known as scleroderma) (3, 4). Unfortunately, PAH is highly morbid and lethal, with 50% of patients dying within 7 years of initial diagnosis (5). Furthermore, the prognosis of SSc-PAH is dramatically worse than that of IPAH (6). Despite a number of available medications for PAH, no therapies induce reversal or prevent progression of the disease. Similarly, among Group 3 patients, PH portends a substantially worse prognosis for the underlying lung disease (3).

Many cardiovascular diseases, such as atherosclerosis and arterial restenosis, are characterized by excess and aberrant smooth muscle cells (SMCs), and similarly SMC coating of normally unmuscularized distal pulmonary arterioles in PH is a key pathological feature. This hypermuscularization reduces pulmonary arterial compliance, which is a strong independent predictor of mortality in IPAH (7). Current treatments for PAH primarily induce vascular dilation, but these therapies do not attenuate the excess muscularization. The treatment gap largely reflects limits in our understanding of pathogenesis, and hence further investigations into the pathobiology of PH are paramount.

We previously found that specialized pulmonary arteriole SMCs expressing platelet-derived growth factor receptor–β (PDGFR-β) clonally expand and give rise to pathological distal arteriole SMCs during hypoxia-induced PH, but regulation of this stereotyped process is incompletely understood (8, 9). Upregulation of hypoxia-inducible factor 1α (HIF1-α) in SMCs plays a key role in distal muscularization, and in addition to such pathways in SMCs themselves, non–cell-autonomous regulation is critical (10, 11). In this context, endothelial cells (ECs) are the most highly studied cell type. For instance, the PDGF pathway is integral to vascular SMC development and disease (12, 13), and deletion of the ligand *Pdgfb* in ECs attenuates hypoxia-induced distal pulmonary arteriole muscularization, PH, and right ventricle hypertrophy (RVH) (11).

Beyond vascular cell types, immune cells, including monocytes/macrophages, have recently received increasing attention in the context of PH (14, 15). With exposure of mice to hypoxia, monocytes migrate to the lung perivascular space and differentiate into interstitial macrophages (16, 17). Bronchoalveolar lavage of these mice demonstrates an increase in macrophages in the aspirated bronchoalveolar lavage fluid (BALF) as well as in the residual lung (18). Similarly, cells expressing the macrophage marker CD68 are enriched in proximity to vascular obstructive lesions in the lungs of human patients with PAH (19). In rodent models of PH, global genetic or pharmacological inhibition of select receptors or agonists expressed by macrophages (e.g., CX3CR1, leukotriene B_4_) has been shown to mitigate PH (18, 20); however, these products are produced by other cell types as well, raising the issue of macrophage specificity.

Although monocytes/macrophages are undoubtedly important players in the pathogenesis of PH and other vascular diseases, their roles in regulating the biology of SMCs in these contexts are not well established. We recently demonstrated that during the formation of atherosclerotic plaques, clonal expansion of rare SMCs is regulated by bone marrow–derived cells (most likely macrophages) (21). Furthermore, medium conditioned by activated macrophages from atheroprone mice induces aortic SMC migration and proliferation (21, 22). Relevant to PH, hypoxia exposure of macrophages preactivated by interleukin-4 generates conditioned medium that induces proliferation of pulmonary artery SMCs (PASMCs) (23). In addition, dual inhibition of C-C motif chemokine receptor 2 and 5 attenuates macrophage-conditioned medium induction of PASMC proliferation and migration (24). Finally, we recently found that downregulation of PDGF-B in monocytes/macrophages with the inefficient *Csf1r-Mer-iCre-Mer* modestly inhibits hypoxia-induced pulmonary vascular remodeling, but hemodynamics and underlying pathways were not assessed (11, 25, 26).

Herein, we present findings establishing that lung macrophage-derived PDGF-B plays a key role in pathological SMC expansion and, thus, PH and has genuine potential as a therapeutic target. To this end, our studies used mouse models, cell type–specific deletion of multiple genes, human macrophages from IPAH and SSc-PAH patients, and in vivo nanoparticle-delivered siRNA against Pdgfb. We find that depletion of lung macrophages or *Pdgfb* deletion in myeloid cells attenuated hypoxia-induced distal muscularization, PH, and alveolar myofibroblast accumulation. Our results indicate that HIF1-α and HIF2-α are upstream of PDGF-B in macrophages and deletion of either *Hifa* gene in LysM^+^ cells in hypoxia-exposed mice has similar effects as *Pdgfb* deletion. As a complementary approach, under normoxic conditions, HIFα gain of function in myeloid cells induced lung macrophage accumulation and Pdgfb expression as well as distal muscularization, PH, and RVH. Medium conditioned by macrophages from IPAH and SSc-PAH patients induced human PASMC (hPASMC) proliferation and migration in a PDGF-B–dependent manner. Finally, our results indicate that orotracheally administered nanoparticles loaded with Pdgfb siRNA markedly attenuated hypoxia-induced lung macrophage Pdgfb expression, distal muscularization, PH, RVH, and alveolar myofibroblast accumulation. Taken together, further evaluation of approaches targeting lung macrophage-derived PDGF-B is of high priority as a strategy to combat PH.

## Results

### Alveolar and parenchymal lung macrophages accumulate in hypoxia, and their depletion attenuates distal muscularization and PH.

As with our prior studies, immunohistochemical analysis of distal muscularization in the investigations herein focused on specific pulmonary arteriole beds adjacent to identified airway branches left bronchus-first lateral secondary branch-first anterior branch-first lateral or first medial branch (L.L1.A1.L1 or L.L1.A1.M1) (8, 9, 11). Under normoxic conditions, distal arterioles in these beds are unmuscularized but undergo a stereotyped process of muscularization with hypoxia exposure (8, 9, 11).

**Figure 1:**
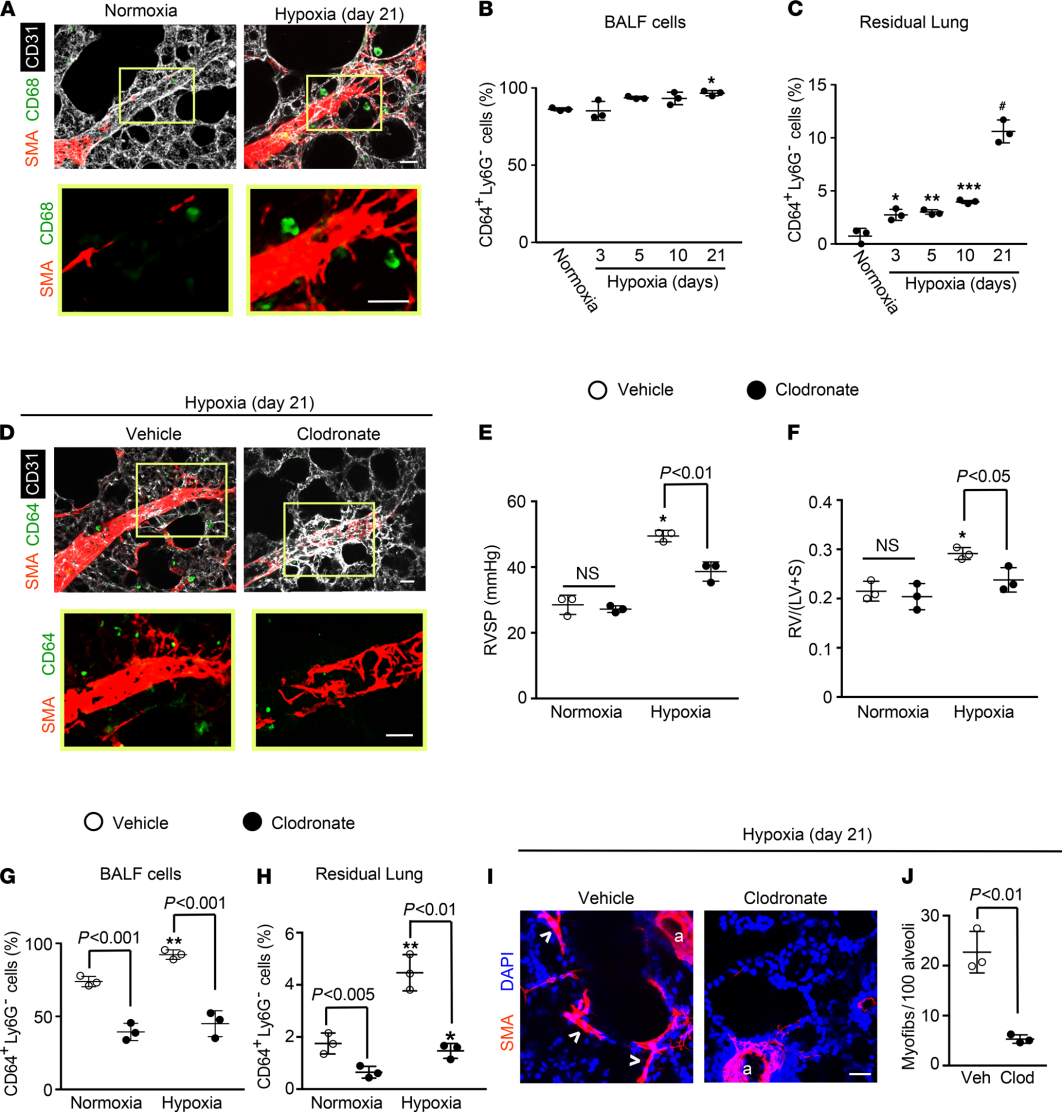
Lung macrophages accumulate with hypoxia and are critical for hypoxia-induced pulmonary vascular remodeling and PH. WT mice were exposed to hypoxia (10% FiO_2_) for up to 21 days or maintained in normoxia as indicated. (**A**) Vibratome sections including distal arterioles of the L.L1.A1 regions of left lung were stained for markers of SMCs (α–smooth muscle actin [SMA]), macrophages (CD68), and ECs (CD31). The boxed region is shown as close-ups below. *n* = 6 mice. (**B** and **C**) BALF and residual lung were harvested, and single-cell suspensions were subjected to flow cytometric analysis. The percentage of total cells in the given compartment that are CD64^+^Ly6G^–^ macrophages was determined. *n* = 3 mice per time point. (**D**–**J**) Liposomes containing PBS (vehicle) or clodronate were administered orotracheally at the onset of hypoxia (or normoxia as a control) and two times per week thereafter during the 21-day treatment. (**D**) Lung vibratome sections of the L.L1.A1.M1 region were stained for SMA, CD64, and CD31 with boxed regions magnified below. *n* = 4–5 mice. RVSP (**E**) and Fulton index (**F**; weight ratio of the right ventricle [RV] to sum of the left ventricle [LV] and septum [S]) are shown. *n* = 3 mice. (**G** and **H**) The percent of CD64^+^Ly6G^–^ macrophages in total cells of the BALF and residual lung was determined. *n* = 3 mice. (**I** and **J**) Alveolar regions were stained for SMA and nuclei (DAPI), and the number of alveolar myofibroblasts (arrowheads) per 100 alveoli was determined. Arterioles are indicated by “a.” *n* = 3 mice. More than 500 alveoli were quantified per mouse. One-way ANOVA with Tukey’s multiple-comparison test (*, **, ***, ^#^ vs. normoxia, *P* < 0.05, < 0.01, < 0.001, < 0.0001, respectively) was used in **B**, **C**, and **E**–**H**, and Student’s *t* test was used in **J**. Scale bars: 25 μm.

In addition to developing distal arteriole muscularization and PH, the lungs of mice exposed to hypoxia accumulate excess macrophages (18, 27, 28) (Figure 1, A–C). We initially determined the time course of lung macrophage accumulation during PH in WT mice maintained in hypoxia (FiO_2_ 10%) for up to 21 days. The pulmonary vasculature was flushed, and then using flow cytometry, we isolated CD64^+^Ly6G^–^ macrophages from BALF and from the residual lung after BALF aspiration (Supplemental Figure 1; supplemental material available online with this article; https://doi.org/10.1172/jci.insight.139067DS1). The percentage of macrophages in BALF gradually increased, reaching statistical significance on hypoxia day 21 in comparison with normoxia (Figure 1B). In contrast, macrophages from the residual lung are 2.9 ± 0.5–fold increased by hypoxia day 3 and up to 10.8 ± 1.1–fold increased at hypoxia day 21 (Figure 1C).

We next evaluated the effects of depletion of alveolar and residual macrophages with clodronate on hypoxia-induced distal muscularization and PH. Liposomes loaded with clodronate or as a control with phosphate-buffered saline (PBS) were administered orotracheally to WT mice at the onset of hypoxia and 2 times per week during the ensuing 21 days of hypoxia to deplete phagocytes. Mice treated with clodronate had attenuated hypoxia-induced distal muscularization, right ventricular systolic pressure (RVSP; equivalent to pulmonary artery systolic pressure), and RVH as measured by the Fulton index (i.e., weight ratio of the right ventricle [RV] to the sum of the left ventricle [LV] and septum [S]) (Figure 1, D–F). In comparison with control liposomes, treatment with clodronate-loaded liposomes reduced macrophages by approximately 50% in the BALF and approximately 65% in the residual lung (Figure 1, G and H). Finally, under basal conditions, the adult lung has very rare myofibroblasts, but we and others have demonstrated that hypoxia induces a marked increase in the number of these cells (8, 29). Herein, we found that depletion of myeloid cells markedly inhibited hypoxia-induced accumulation of alveolar SMA^+^ myofibroblasts (Figure 1, I and J).

### Lung macrophage Pdgfb is upregulated with hypoxia, and Pdgfb deletion in the LysM^+^ or CSF1R^+^ cells attenuates PH.

**Figure 2:**
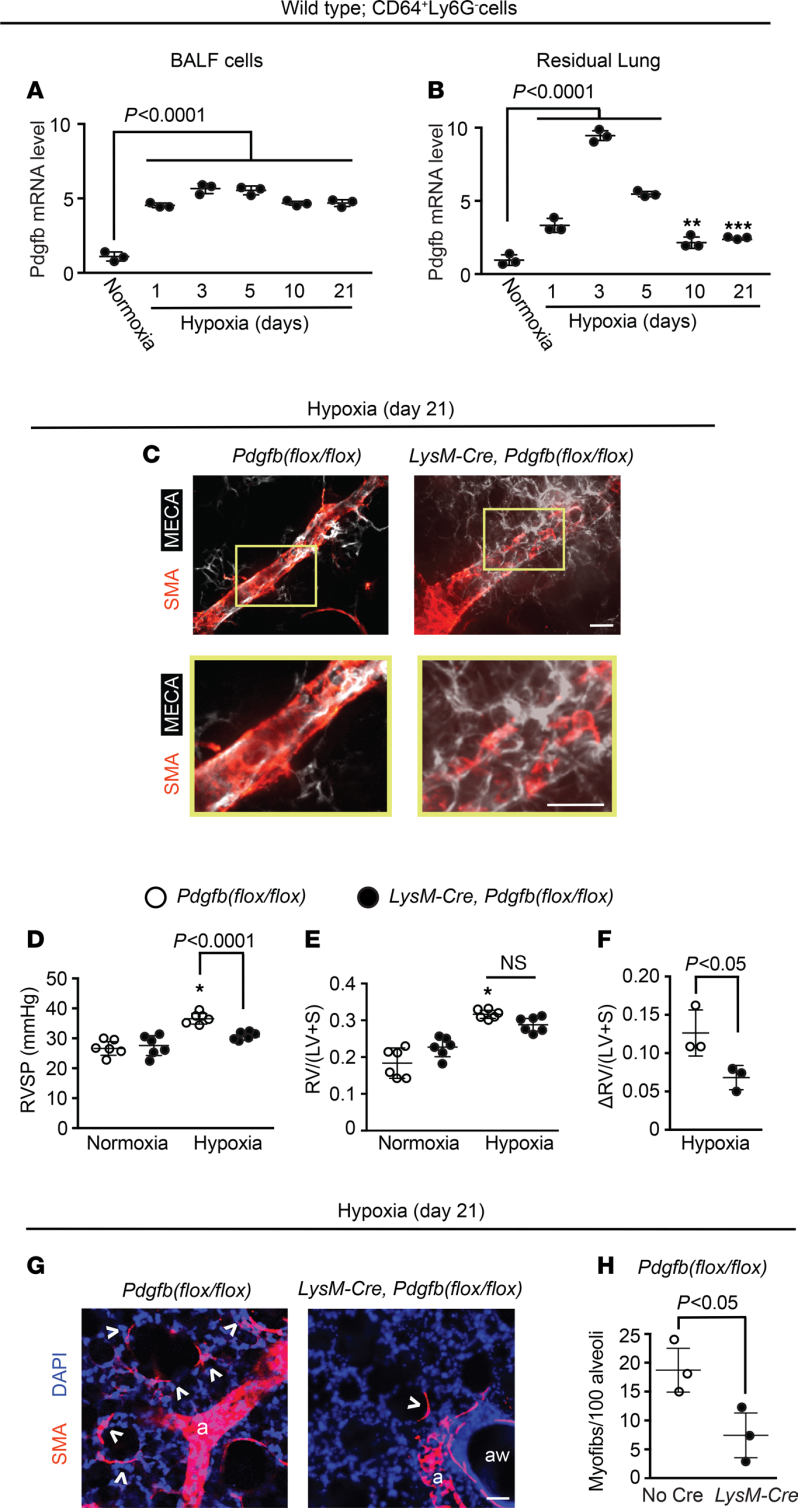
Lung macrophage Pdgfb levels increase with hypoxia, and *Pdgfb* deletion in LysM^+^ cells attenuates distal muscularization and PH. (**A** and **B**) BALF and residual lung CD64^+^Ly6G^-^ cells were isolated by FACS from WT mice exposed to hypoxia (10% FiO_2_) for up to 21 days or normoxia as indicated. *Pdgfb* mRNA levels were measured by qRT-PCR (see Supplemental Table 1). *n* = 3 mice per time point with qRT-PCR done in triplicate. (**C**–**H**) *Pdgfb^fl/fl^* mice also carrying no Cre or *LysM-Cre* were exposed to hypoxia for 21 days or maintained in normoxia. (**C**) Vibratome sections with distal arterioles of the L.L1.A1 lung regions were stained for SMA and EC marker MECA-32. Boxed regions are shown below as close-ups. *n* = 6 mice. RVSP and Fulton index measurements are shown (**D** and **E**). *n* = 6 mice. In addition, the Fulton index differences between hypoxia and normoxia values stratified by genotype are displayed (**F**). (**G** and **H**) Alveolar regions were stained for SMA and nuclei (DAPI), and the number of alveolar myofibroblasts (arrowheads) per 100 alveoli was determined. Arterioles and airways are indicated by “a” and “aw,” respectively. *n* = 3 mice; more than 500 alveoli were quantified per mouse. One-way ANOVA with Tukey’s multiple-comparison test (*, **, *** vs. normoxia, *P* < 0.05, < 0.01, < 0.001, respectively) was used in **A**, **B**, **D**, and **E**, and Student’s *t* test was used in **F** and **H**. Scale bars: 25 μm.

Exposure of mice to hypoxia increases PDGF-B levels in the whole lung and in lung ECs specifically (9, 11); however, not all lung PDGF-B derives from ECs (9, 11, 30, 31). Thus, we analyzed a time course of Pdgfb expression in CD64^+^Ly6G^–^ macrophages isolated by FACS from the BALF and residual lung of mice exposed to hypoxia for up to 21 days. *Pdgfb* mRNA level was measured by quantitative real-time PCR (qRT-PCR) (Supplemental Table 1) and in comparison with normoxia, was increased within 1 day of hypoxia and peaked at day 3 at a level of 5.6 ± 0.2–fold and 9.3 ± 0.2–fold increase for BALF and residual lung, respectively (Figure 2, A and B). Additionally, there was a 2.5 ± 0.4–fold increase in PDGF-B protein in the BALF at hypoxia day 3 compared with normoxia (Supplemental Figure 2). To further confirm the upregulation of Pdgfb in monocytes/macrophages, we used LysM-Cre, which marks this population (32). *LysM-Cre ROSA26R^mTmG/mTmG^* mice were exposed to hypoxia for 21 days or maintained in normoxia, and then GFP^+^ cells were isolated by FACS from whole lung. *Pdgfb* mRNA level was increased by 2.1 ± 0.4–fold in cells isolated from hypoxic mice (Supplemental Figure 3A). Similarly, GFP^+^ cells isolated from BALF of normoxic mice had increased *Pdgfb* mRNA levels when cultured under hypoxic (3% O_2_) as opposed to normoxic conditions (Supplemental Figure 3B).

We next evaluated whether monocyte/macrophage-derived PDGF-B contributes to hypoxia-induced PH. Previously, we found that tamoxifen treatment of *Csf1r-Mer-iCre-Mer Pdgfb^fl/fl^* mice modestly attenuates pathological distal pulmonary arteriole muscularization (11), but effects on PH, RVH, and myofibroblast accumulation were not studied. Given the inefficiency of this Cre for inducing recombination (25, 26), in the current studies, *Pdgfb^fl/fl^* mice carrying *Csf1r-Mer-iCre-Mer* or no Cre were injected with tamoxifen for 15 days (1 mg/d). Mice were then rested for 5 days and subsequently exposed to hypoxia for 21 days. In addition to inhibiting hypoxia-induced distal muscularization, *Pdgfb* deletion in CSF1R^+^ cells inhibited RVSP and the Fulton index (Supplemental Figure 4). To bypass the inefficiency of the inducible Csf1r-Cre, further studies used the constitutive LysM-Cre to delete *Pdgfb* (Supplemental Figure 5A). Importantly, LysM-Cre–mediated *Pdgfb* deletion did not alter Pdgfb levels in lung ECs (Supplemental Figure 5B). On the *Pdgfb^fl/fl^* background, mice also carrying *LysM‑Cre* had attenuated distal muscularization and PH with 21-day hypoxia exposure in comparison with those with no Cre (Figure 2, C and D). When comparing the Fulton index of *LysM-Cre Pdgfb^fl/fl^* with that of *Pdgfb^fl/fl^* mice, there was a trend toward reduction with hypoxia and increase with normoxia, but these differences did not reach statistical significance (Figure 2E). However, when the Fulton index differences between hypoxia and normoxia values were stratified by genotype, there was a significant 46% ± 7% reduction in this difference for *LysM-Cre Pdgfb^fl/fl^* mice (Figure 2F). Finally, with myeloid cell *Pdgfb* deletion, myofibroblasts were reduced by approximately 60% at both 3 and 21 days of hypoxia (Figure 2, G and H, and Supplemental Figure 5, C and D). Thus, myeloid cell–derived PDGF-B is an important player in hypoxia-induced pulmonary vascular remodeling and PH.

### LysM-Cre–mediated deletion of von-Hippel Lindau induces Pdgfb expression and pulmonary vascular remodeling in normoxia.

**Figure 3:**
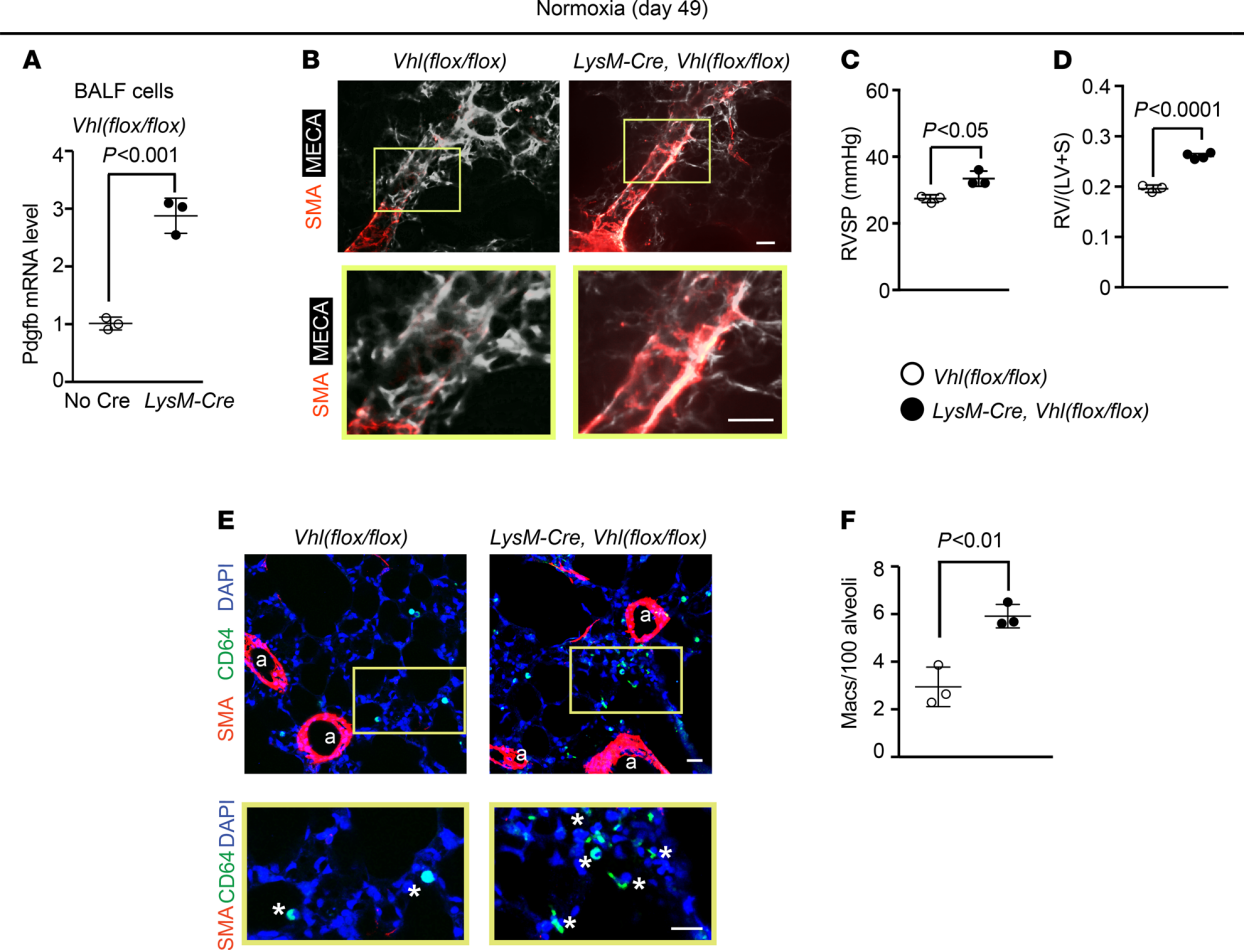
*Vhl* deletion in LysM^+^ cells induces distal muscularization and PH under normoxia. *Vhl^fl/fl^* mice also carrying no Cre or *LysM-Cre* were maintained in normoxia for 49 days after birth. (**A**) BALF was isolated and Pdgfb transcript levels were measured by qRT-PCR. (**B**) Lung vibratome sections of L.L1.A1.L1 region were stained for SMA and MECA-32 with boxed regions magnified below. RVSP (**C**) and Fulton index (**D**) are shown. (**E**) Lung vibratome sections were stained for SMA, CD64, and nuclei (DAPI), with arterioles labeled with “a.” Boxed regions shown are shown below as close-ups, and the number of macrophages (asterisks) quantified per 100 alveoli (**F**). More than 500 alveoli per mouse were quantified. *n* = 3 mice. Student’s *t* test was used in **A**, **C**, **D**, and **F**. Scale bars: 25 μm.

Given the critical role of myeloid cell–derived PDGF-B in the pathogenesis of PH, we next endeavored to evaluate mechanisms underlying hypoxia-induced Pdgfb expression by this cell type. HIFs are heterodimers of HIF1-β and a HIFα isoform, either HIF1-α or HIF2-α. In mice exposed to hypoxia, EC HIF regulates cell-autonomous Pdgfb expression as well as distal muscularization and PH (11, 33, 34). Using oxygen as a substrate, HIFα undergoes proline hydroxylation, a modification that facilitates binding to von Hippel Lindau–E3 (VHL-E3) ubiquitin ligase and ultimately proteosome‑mediated degradation (35). Thus, HIFα accumulates when oxygen is scare or when the relevant ubiquitination-degradation pathway is inhibited, such as by *Vhl* deletion (11, 36). Under normoxic conditions, in comparison with *Vhl^fl/fl^* mice, *LysM-Cre Vhl^fl/fl^* mice had reduced *Vhl* and increased *Hif1a*, *Hif2a*, and *Pdgfb* mRNA levels in BALF cells and increased PDGF-B protein in BALF (Figure 3A and Supplemental Figure 6). Furthermore, *Vhl* deletion in myeloid cells induced distal muscularization, PH, and RVH in normoxia (Figure 3, B–D) as well as lung macrophage accumulation (Figure 3, E and F).

We then evaluated whether *Vhl* deletion potentiates the effects of a relatively brief (7-day) exposure to hypoxia. At this time point, *Vhl^fl/fl^* mice carrying *LysM-Cre* had BALF cell *Pdgfb* mRNA levels that were robustly increased at 7.6 ± 1.2–fold relative to those of mice lacking Cre (Supplemental Figure 7A). Furthermore, *Vhl* deletion in LysM^+^ cells induced markedly enhanced distal muscularization as well as increased RVSP and RVH following brief hypoxia exposure (Supplemental Figure 7, B–D).

### Myeloid cell HIFα regulates Pdgfb expression and hypoxia-induced distal muscularization, RVH, and PH.

**Figure 4:**
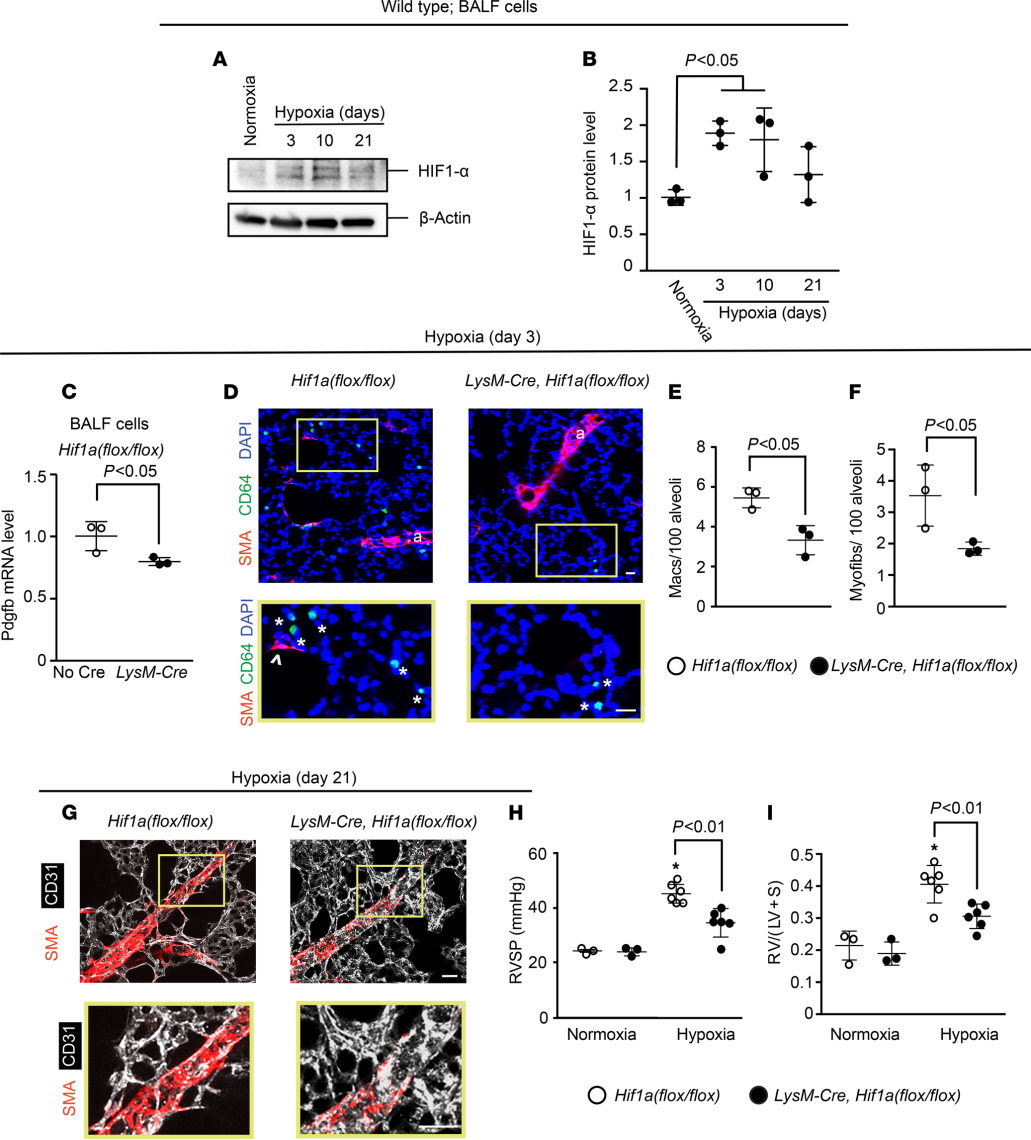
*Hif1a* deletion in myeloid cells attenuates hypoxia-induced Pdgfb expression, distal muscularization, and PH. (**A** and **B**) BALF cells were isolated from normoxic or hypoxic (10% FiO_2_ up to 21 days) WT mice. HIF1-α and β-actin protein were assessed by Western blot (**A**) with densitometry of HIF1-α relative to β-actin (**B**). *n* = 3 mice per time point. (**C**–**I**) *Hif1a^fl/fl^* mice also carrying no Cre or *LysM-Cre* were exposed to hypoxia for 3 or 21 days. At hypoxia day 3, Pdgfb transcript levels of BALF cells were determined by qRT-PCR (**C**). Lung vibratome sections were stained for SMA, macrophage marker CD64, and nuclei (DAPI) with arterioles indicated by “a” and boxed regions shown as close-ups below (**D**). The numbers of macrophages (asterisks) and alveolar myofibroblasts (arrowhead) were quantified per 100 alveoli (**D**–**F**). *n* = 3–5 mice; qRT-PCR was done in triplicate. More than 700 alveoli were quantified per mouse. At hypoxia day 21, vibratome sections with distal arterioles in the L.L1.A1.L1 area were stained for SMA and CD31 (**G**), and RVSP and the Fulton index were measured as shown (**H** and **I**). *n* = 3 mice. One-way ANOVA with Tukey’s multiple-comparison test was used in **B**, **H**, and **I** (* vs. normoxia, *P* < 0.05), and Student’s *t* test was used in **C**, **E**, and **F**. Scale bars: 25 μm.

**Figure 5:**
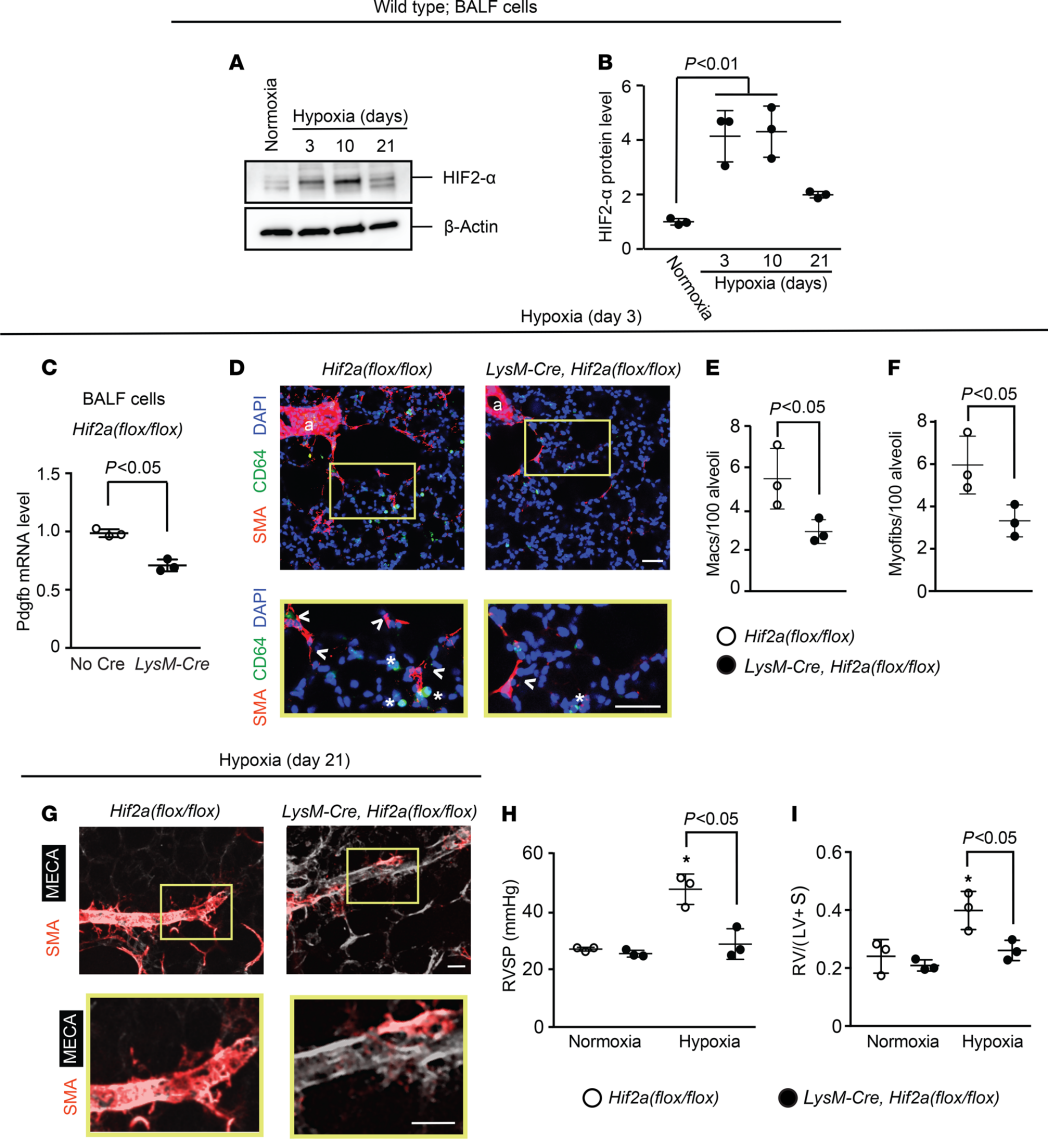
Deletion of *Hif2a* in LysM^+^ cells attenuates hypoxia-induced Pdgfb expression, distal muscularization, and PH. (**A** and **B**) BALF cells were isolated from WT mice exposed to normoxia or hypoxia (10% FiO_2_) for up to 21 days. Western blot was used to assess HIF2-α and β-actin protein levels (**A**) with densitometry of HIF2-α relative to β-actin (**B**). *n* = 3 mice per time point. (**C**–**I**) *Hif2a^fl/fl^* mice also carrying no Cre or *LysM-Cre* were exposed to hypoxia for 3 or 21 days. At hypoxia day 3, BALF cells were isolated, with *Pdgfb* mRNA levels determined by qRT-PCR (**C**), and vibratome sections of the lung were stained for SMA, CD64, and nuclei (DAPI) with arterioles indicated by “a” and with close-ups of the boxed regions shown below (**D**). The numbers of macrophages (asterisks) and alveolar myofibroblasts (arrowheads) were quantified per 100 alveoli (**D**–**F**). *n* = 3–5 mice; qRT-PCR was done in triplicate. More than 700 alveoli were quantified per mouse. At hypoxia day 21, vibratome sections with distal arterioles in the L.L1.A1.L1 area were stained for SMA and MECA-32 (**G**), and RVSP and the Fulton index were measured (**H** and **I**). *n* = 3 mice. One-way ANOVA with Tukey’s multiple-comparison test was used in **B**, **H**, and **I** (* vs. normoxia, *P* < 0.05), and Student’s *t* test was used in **C**, **E**, and **F**. Scale bars: 50 μm (**D**) and 25 μm (**G**).

To complement the experiments that deleted *Vhl* and, thus, induced the HIF pathway, we next pursued studies that deleted *Hif1a* or *Hif2a* in LysM^+^ cells. First, a time course of hypoxia exposure of WT mice revealed HIF1-α upregulation in BALF cells by hypoxia day 3 (Figure 4, A and B). At this time point, mice on the *Hif1a^fl/fl^* background and carrying *LysM-Cre* had reduced levels of Pdgfb and Hif1a in BALF cells in comparison with mice lacking Cre (Figure 4C and Supplemental Figure 8A). In addition, accumulation in the lung of cells expressing the macrophage marker CD64 and of myofibroblasts was substantially reduced with *Hif1a* deletion (Figure 4, D–F). Moreover, analysis at hypoxia day 21 revealed that *LysM-Cre Hif1a^fl/fl^* mice had attenuated distal pulmonary arteriole muscularization, RVSP, and Fulton index (Figure 4, G–I). Findings were similar in regard to both hypoxia-induced HIF2-α levels in BALF cells and the effects of *Hif2a* deletion on the lung phenotype of hypoxic *LysM-Cre* *Hif2a^fl/fl^* mice (Figure 5 and Supplemental Figure 8B). Thus, taking *Pdgfb*, *Vhl*, *Hif1a*, and *Hif2a* deletion experiments together, the results suggest that PDGF-B expression by myeloid cells is modulated cell autonomously by both HIFα isoforms and is a key factor regulating pulmonary vascular remodeling and PH.

### Macrophage-derived PDGF-B is increased in PAH patients and induces SMC proliferation and migration.

**Figure 6:**
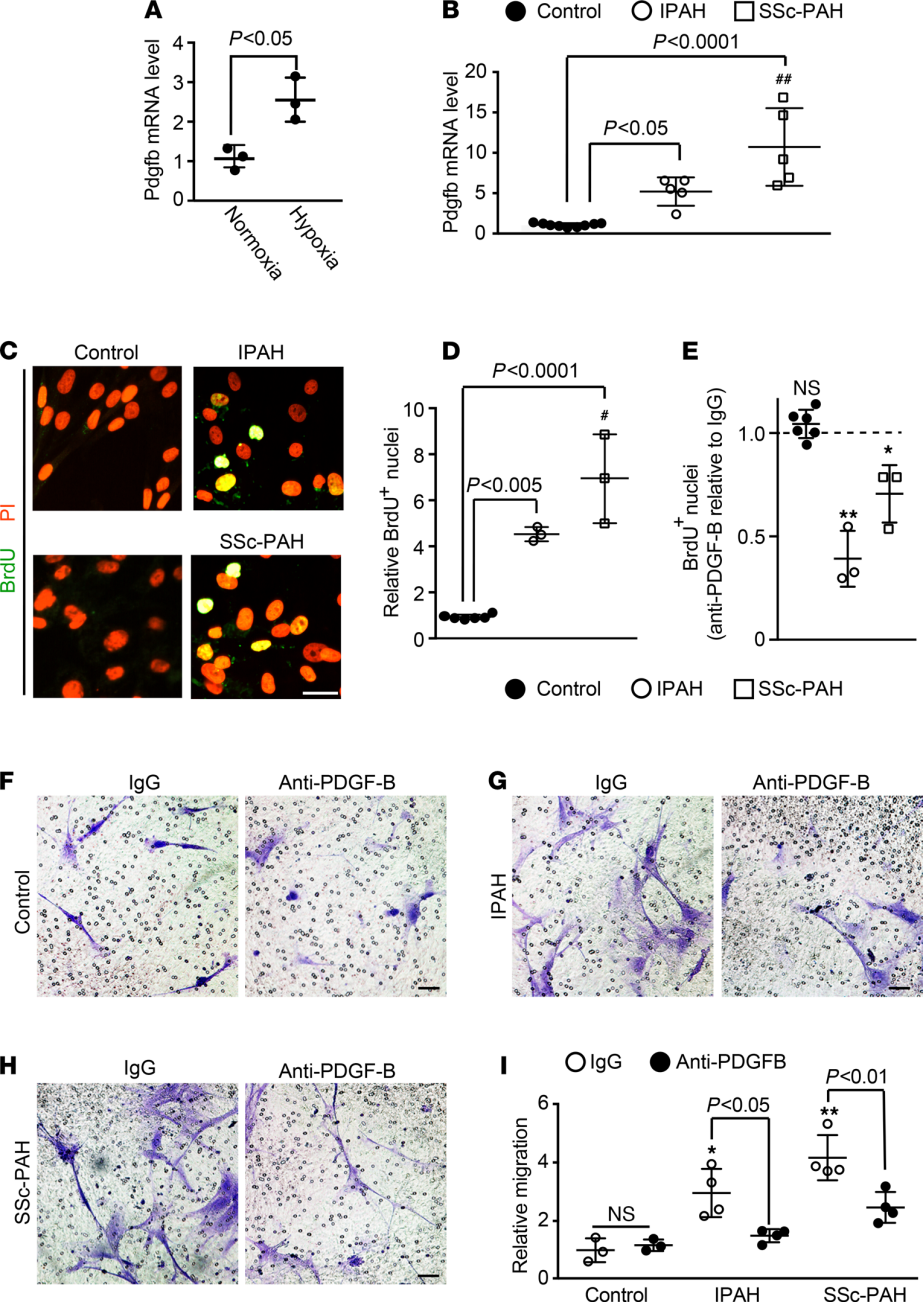
PDGF-B secreted by macrophages from PAH patients promotes hPASMC proliferation and migration. Monocytes were isolated from PBMCs of human controls and IPAH or SSc-PAH patients and differentiated into macrophages in culture. (**A**) Macrophages derived from human control monocytes were cultured under normoxic or hypoxic (3% O_2_) conditions for 12 hours, and then *Pdgfb* mRNA levels were measured by qRT-PCR. *n* = 3 humans (2 females and 1 male, aged 30–60 years old) with qRT-PCR done in triplicate. (**B**) qRT-PCR was used to assay *Pdgfb* mRNA levels of macrophages from controls and PAH patients. *n* = 5 humans per PAH diagnostic class, and *n* = 9 controls (see Supplemental Table 4), with qRT-PCR done in triplicate. (**C**–**E**) hPASMCs were cultured for 24 hours with medium preconditioned by control and patient macrophages. BrdU was included in the last 10 hours of this incubation. Cells were then stained for BrdU and nuclei (PI). (**D**) The percentage of total cells (PI^+^ nuclei) expressing BrdU for control humans and patients was normalized to this percentage for controls. (**E**) Anti–PDGF-B blocking antibody or control IgG was added to the conditioned medium 1 hour prior to incubation with hPASMCs. Results are the ratio of the percentage of total (PI^+^) cells that are BrdU^+^ for anti–PDGF-B treatment relative to IgG treatment, stratified by patient diagnostic class. *n* = 3 humans per PAH diagnostic class and *n* = 6 controls (see Supplemental Table 5), with 10 microscopic fields per human, 30–60 cells per field. (**F**–**I**) Medium preconditioned by control or patient macrophages was treated with anti–PDGF-B blocking or control IgG antibody for 1 hour and then placed in the bottom chamber of a Boyden apparatus. hPASMCs were added to the top chamber to assess migration toward the conditioned medium for 8 hours. Migrated cells (i.e., on the membrane’s bottom surface) were stained with Crystal Violet. (**I**) Quantification of the migrated cells relative to control patients, IgG treatment is shown. *n* = 4 humans per PAH class and *n* = 3 controls (see Supplemental Table 7), with 5 microscopic fields per human, 8–90 cells per field. One-way ANOVA with Tukey’s multiple-comparison test (**B**, **D**, and **I**) and Student’s *t* test were used (**A** and **E**). ^#^, ^##^ vs. IPAH, *P* < 0.05, <0.01, and *, **, ns vs. corresponding IgG controls, *P* < 0.05, < 0.01, not significant, respectively. Scale bars: 25 μm (**C**) and 50 μm (**F**–**H**).

Given the prominent role of macrophages and myeloid cell–derived PDGF-B in pathological lung muscularization in mice, we next sought to extrapolate these findings to human patients with PAH (Supplemental Tables 2 and 3). Initially, Pdgfb levels from human macrophages were analyzed. The peripheral blood mononuclear cell (PBMC) fraction was isolated from fresh whole blood of control humans by Ficoll column centrifugation and enriched for monocytes by adherence to plastic (37, 38) (Supplemental Figure 9, A and B). Adherent cells were incubated with macrophage colony-stimulating factor to differentiate them to macrophages (38), and exposure of macrophages to hypoxia (3% O_2_) as opposed to normoxia for 12 hours induced a 2.6 ± 0.6–fold increase in *Pdgfb* mRNA (Figure 6A). As strong evidence of the clinical relevance of this work, Pdgfb levels of macrophages differentiated from circulating monocytes of IPAH and SSc-PAH patients were enhanced by 5.1 ± 1.8–fold and 10.7 ± 4.8–fold, respectively, in comparison with those of control humans (Figure 6B and Supplemental Table 4). Furthermore, PDGF-B protein was increased in medium conditioned by macrophages from these patients with PAH compared with controls (Supplemental Figure 9C).

We then evaluated the effect of medium conditioned by macrophages from patients with PAH on hPASMC proliferation and the role of PDGF-B in this medium. hPASMCs were cultured for 24 hours in medium conditioned by newly differentiated macrophages, and BrdU was added for the final 10 hours of this incubation. The percentage of cells (PI^+^ nuclei) that were proliferative (i.e., BrdU^+^) relative to control was determined (Figure 6, C and D, and Supplemental Table 5). For medium conditioned by macrophages derived from IPAH and SSc-PAH patients, there was a relative increase in hPASMC proliferation by 4.6 ± 0.3–fold and 7.0 ± 1.9–fold, respectively. To evaluate the contribution of PDGF-B to these effects, macrophage-conditioned medium was incubated with anti–PDGF-B blocking antibody or IgG control for 1 hour prior to adding to hPASMCs. For macrophages derived from control patients, hPASMC proliferation was not changed by anti–PDGF-B pretreatment whereas this pretreatment significantly inhibited hPASMC proliferation induced by medium conditioned by IPAH or SSc-PAH macrophages (Figure 6E and Supplemental Figure 9D). A qualitatively similar — albeit less robust — increase in hPASMC proliferation occurred with exposure to medium conditioned by macrophages that were generated from cryopreserved PBMCs of patients with PAH (Supplemental Figure 9E and Supplemental Table 6). As with the studies with fresh PBMCs, anti–PDGF-B treatment markedly inhibited hPASMC proliferation in medium conditioned by macrophages from cryopreserved PAH, but not control, PBMCs (Supplemental Figure 9F).

Next, a similar approach was used to investigate the effect of medium conditioned by macrophages (from fresh PBMCs) and PDGF-B therein on hPASMC migration. We assessed hPASMC migration from the top of a Boyden chamber toward the bottom chamber containing conditioned medium pretreated, as in the proliferation studies, with an anti–PDGF-B or IgG control antibody. For IgG control pretreatment, conditioned medium from IPAH or SSc-PAH macrophages induced migration relative to that from control macrophages by 3.0 ± 0.8–fold or 4.2 ± 0.8–fold, respectively (Figure 6, F–I, and Supplemental Table 7). Furthermore, in comparison with IgG pretreatment, anti–PDGF-B pretreatment reduced hPASMC migration with IPAH or SSc-PAH macrophage-conditioned medium by approximately 40%–50%. In contrast, anti–PDGF-B pretreatment of conditioned medium from control humans did not affect hPASMC migration.

### Nanoparticle delivery of siRNA targeting Pdgfb attenuates hypoxia-induced PH.

**Figure 7:**
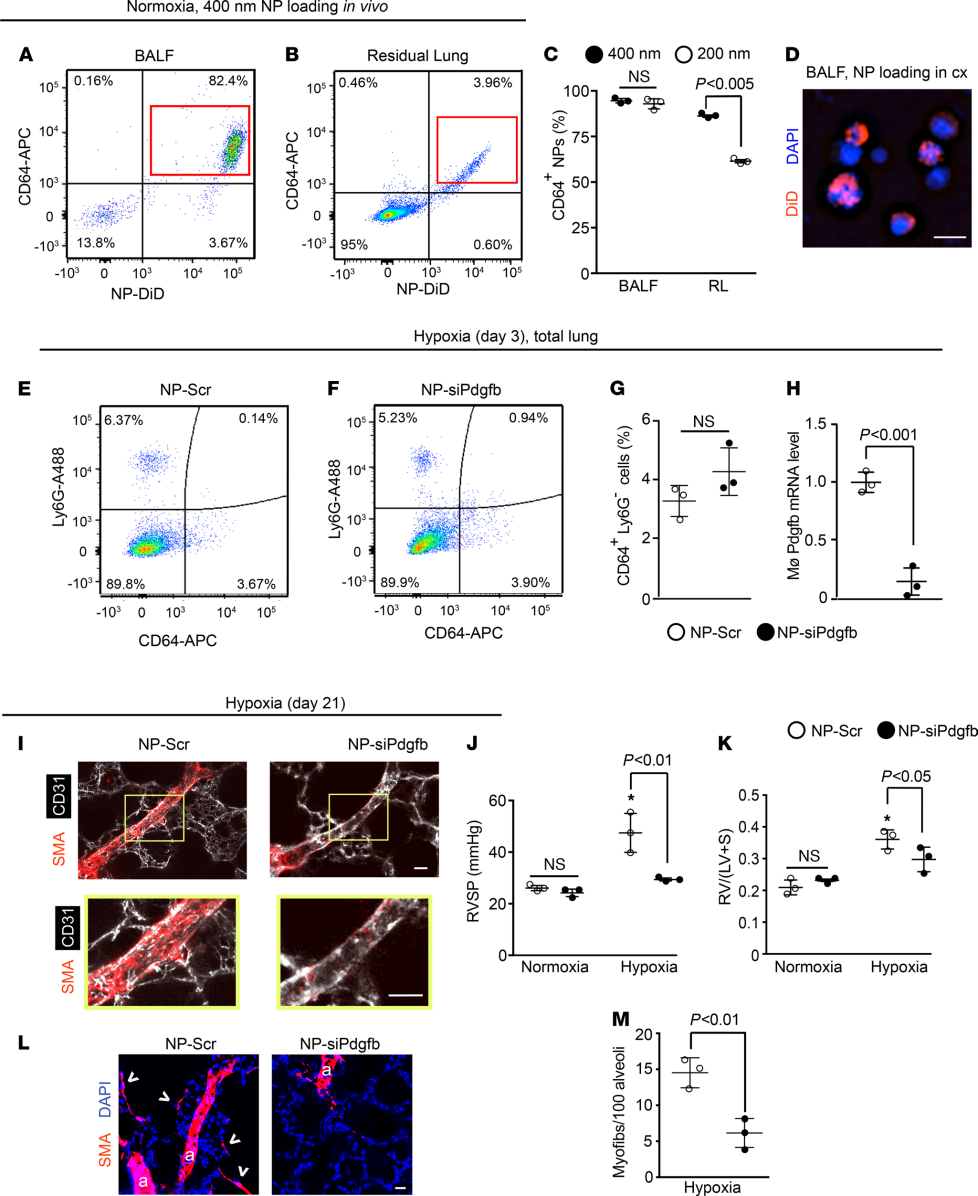
Nanoparticle-mediated knockdown of Pdgfb attenuates distal arteriole muscularization, myofibroblast accumulation, and PH. (**A** and **B**) Nanoparticles (diameter 400 nm) loaded with the dye DiD were administered orotracheally to normoxic mice, and 12 hours later, cells from BALF and residual lung were stained for CD64 and subjected to flow cytometric analysis. (**C**) Quantification of experiments from **A** and **B** and Supplemental Figure 10, A and B, showing percentage of BALF or residual lung (RL) cells containing DiD^+^ nanoparticles (diameter 400 or 200 nm as indicated) expressing CD64. *n* = 3 mice per treatment. (**D**) BALF cells were harvested from normoxic mice, cultured with DiD-loaded 400 nm nanoparticles for 6 hours, and then stained for nuclei (DAPI). (**E**–**M**) Nanoparticles of 400 nm diameter were loaded with siRNA targeted against Pdgfb or scrambled (Scr) RNA, then administered to mice at the onset of hypoxia and twice per week thereafter. (**E** and **F**) Lungs were isolated from mice at hypoxia day 3, stained for Ly6G and CD64, and subjected to flow cytometry, and the percentage of CD64^+^Ly6G^–^ macrophages was quantified (**G**). *n* = 3 mice per treatment. (**H**) Pdgfb RNA levels of CD64^+^Ly6G^–^ macrophages isolated as in **E** and **F** were quantified by qRT-PCR. *n* = 3 mice per treatment with qRT-PCR done in triplicate. (**I–M**) Mice were treated with hypoxia for 21 days or maintained in normoxia. For hypoxic mice, sections containing distal arterioles in the L.L1.A1 area (**I**) or alveolar region (**L**) were stained for CD31 and SMA. Boxed regions are shown below as close-ups. RVSP (**J**), Fulton index (**K**), and number of myofibroblasts (arrowheads) per 100 alveoli were measured. More than 500 alveoli per mouse were quantified. Arterioles are labeled with “a” in **L**. *n* = 3 mice per treatment group. One-way ANOVA with Tukey’s multiple-comparison test (**C**, **J**, and **K**) and Student’s *t* test were used (**G**, **H**, and **M**). * vs. normoxia, *P* < 0.05. Scale bars: 10 μm (**D**) and 25 μm (**I** and **L**).

After demonstrating the importance of myeloid-derived PDGF-B in experimental PH and the inductive effects of PDGF-B from macrophages of PAH patients on hPASMCs, we next aimed to pharmacologically downregulate this ligand in lung macrophages by delivering nanoparticles formed from a poly(amine-co-ester) (PACE) polymer and *Pdgfb* siRNA. In prior studies, we have shown that similar nanoparticles are capable of sustained silencing of protein expression in cells that internalize the particles (39). First, 400 nm or 200 nm diameter nanoparticles composed of acid-ended poly(pentadecalactone-co-n-methyldiethanolamineco-sebacate) with 50% lactone (PPMS-50COOH) loaded with the dye DiD were orotracheally administered to WT mice, and 12 hours later, flow cytometric analysis was used to evaluate the uptake by lung cells expressing the macrophage marker CD64 (Figure 7, A and B, and Supplemental Figure 10, A and B). For both 400 and 200 nm diameter nanoparticles, the vast majority of CD64^+^ cells were DiD labeled (>99% in BALF and approximately 92% in residual lung; Supplemental Figure 10C). Similarly, the percentage of DiD-labeled cells that were CD64^+^ was high and equivalent for 400 and 200 nm diameter particles (95% ± 1% and 93% ± 3%, respectively) in BALF; however, in the residual lung, these percentages were 86% ± 1% for 400 nm particles and dropped down to 62% ± 1% for 200 nm particles (Figure 7C). To confirm uptake, isolated BALF cells were cultured with DiD-loaded 400 nm nanoparticles for 6 hours, and these cells displayed perinuclear fluorescence (Figure 7D). Additionally, orotracheal delivery of 400 nm DiD-nanoparticles twice per week for 3 weeks neither affected lung mechanics and histology nor led to uptake by other organs, such as the heart and liver (Supplemental Figure 10, D–G, and Supplemental Figure 11). Thus, all further experiments were conducted with 400 nm diameter nanoparticles.

We then evaluated whether nanoparticles loaded with siRNA targeting *Pdgfb* ameliorated the effects of hypoxia exposure on the murine lung. A *Pdgfb* siRNA oligonucleotide (siPdgfb) was used that when transfected into BALF cells reduced Pdgfb levels by 91% ± 1% in comparison with scrambled (Scr) RNA treatment (Supplemental Figure 12A). Nanoparticles loaded with this siPdgfb or Scr RNA were administered orotracheally at the onset of hypoxia and twice per week for up to 21 days of hypoxia exposure. At hypoxia day 3 or 21, the percentage of cells in the whole lung that were CD64^+^LysG^–^ macrophages did not differ between mice treated with the 2 nanoparticle types (Figure 7, E–G, and Supplemental Figure 12, B–D). We then assayed the effect of siPdgfb nanoparticles on macrophage Pdgfb RNA levels at day 3, the time of maximal Pdgfb levels (see Figure 2, A and B). Nanoparticles loaded with siPdgfb reduced lung macrophage Pdgfb levels by 86% ± 11% (Figure 7H). Furthermore, siPdgfb nanoparticle treatment during 21-day hypoxia exposure markedly attenuated distal pulmonary arteriole muscularization, PH, RVH, and accumulation of myofibroblasts (Figure 7, I–M) but did not reverse already well established pulmonary vascular remodeling during chronic hypoxia (Supplemental Figure 13). Of note, in the more severe pulmonary vascular disease model of adding weekly Sugen 5416 injections to 21 days of hypoxia, concomitant siPdgfb nanoparticle treatment led to modestly reduced distal muscularization, a trend toward slightly lower RVSP that did not reach statistical significance (reduced by 5.2 ± 3.0 mmHg; *P* = 0.18), and a significantly decreased Fulton index (Supplemental Figure 14).

## Discussion

Expansion of the SMC lineage is increasingly recognized as a key factor in diverse cardiovascular diseases (40); however, in these pathological contexts as well as during normal vascular development, our understanding of the non–cell-autonomous regulation of SMCs by cell types beyond ECs is rudimentary. Phagocytes, including macrophages, play fundamental roles in both the innate immune system and the pathogenesis of many cardiovascular pathologies, including PH. During the embryonic period, fetal macrophage precursors are recruited to the normal lung and differentiate into macrophages, and subsequently, these resident macrophages are maintained by local proliferation. In contrast, during PH, increased monocytes are found in the pulmonary vasculature and perivascular regions and give rise to lung macrophages (16, 17). Although vascular SMCs and lung macrophages are undoubtedly important cell types in PH, a critical unresolved issue is whether and how lung macrophages regulate SMCs in this context. Herein, our studies with mouse models of PH and human macrophages from IPAH and SSc-PAH patients demonstrate that macrophage-derived PDGF-B induces pathological SMC expansion and PH and, thereby, establish macrophage-derived PDGF-B as a key factor in this paradigm. Moreover, our findings with nanoparticle-derived *Pdgfb* siRNA demonstrate an intriguing approach to prevent this disease.

Intratracheally administered clodronate-containing liposomes has previously been shown to deplete alveolar macrophages and reduce hypoxia-induced PH and RVH in rats (41). Herein, we demonstrate that such treatment in mice reduces macrophages in the residual lung, which includes interstitial macrophages, and in the BALF (alveolar macrophages) and also attenuates distal muscularization and hemodynamic changes (Figure 1). Although this approach is beneficial in the short term, chronically depleting macrophages is not feasible given their integral role in innate immunity. Thus, a preferred strategy is to target specific macrophage-derived gene products.

Along these lines, PDGF is widely implicated in the pathogenesis of PH. In human IPAH, mRNA levels of ligands *PDGFA* and *PDGFB* and receptors *PDGFRA* and *PDGFRB* are upregulated in small pulmonary vessels, and PDGFR-β protein is increased in whole lung lysates (42, 43). Mice with a knock-in mutant *Pdgfrb* encoding a protein that is defective in mediating downstream PI3K and PLC-γ signaling have blunted hypoxia-induced pulmonary vascular remodeling, PH, and RVH (43). In a fetal lamb model in which PH is induced by intrauterine partial ligation of the ductus arteriosus, infusion of an anti–PDGF-B aptamer into the pulmonary artery reduces the severity of pulmonary vascular remodeling by one-half and RVH by two-thirds (44). Moreover, global *Pdgfb^+/–^* mice lack hypoxia-induced distal pulmonary arteriole SMCs whereas EC-specific deletion of *Pdgfb* reduces but does not entirely prevent distal muscularization (9, 11).

Herein, we demonstrate that upon exposing mice to hypoxia, expression of *Pdgfb* by alveolar and residual lung macrophages was markedly upregulated (by hypoxia day 3), and *Pdgfb^fl/fl^* mice also carrying *LysM-Cre* or *Csf1r-Mer-iCre-Mer* has substantially attenuated distal muscularization and RVSP (Figure 2 and Supplemental Figures 2 and 4). Additionally, hypoxic *Csf1r-Mer-iCre-Mer, Pdgfb^fl/fl^* mice had a reduced Fulton index. Given that it is constitutive, the LysM-Cre induces more efficient recombination than the inducible Csf1r-Cre; however, LysM-Cre is broadly expressed in myeloid cells (32). Interestingly, in hypoxic *LysM-Cre* *Pdgfb^fl/fl^* mice, there was a trend to a reduction in RVH, but it did not reach statistical significance likely because of a trend toward increased Fulton index under normoxia in these mutants. Indeed, the hypoxia-induced increase in RVH stratified by genotype was reduced by approximately 50% with *Pdgfb* deletion. The explanation for the trend toward enhanced Fulton index under basal conditions in *LysM-Cre* *Pdgfb^fl/fl^* mice is not clear, but we speculate that myeloid cell–derived PDGF-B may limit RV mass during normal development and/or maintenance.

The aforementioned data indicate that lung macrophage-derived PDGF-B plays an important role in PH; however, the regulation of PDGF-B expression in this cell type is poorly understood. With exposure of mice to hypoxia, lung ECs increased *Pdgfb* levels in a HIF1-α–dependent manner (11), and herein, we found that myeloid cell *Hif1a* or *Hif2a* deletion reduced *Pdgfb* levels in lung macrophages compared with control mice (Figure 4 and Figure 5). Our data indicate that *Hif1a* deletion in myeloid cells is protective against hypoxia-induced PH, which is in agreement with a recent study (45). In addition, *LysM-Cre Hif2a^fl/fl^* mice are protected from Schistosoma-induced PH (46), and our results indicate that these mice similarly have attenuated hypoxia-induced PH. Our complementary HIF gain-of-function studies (i.e., myeloid *Vhl* deletion) suggest that lung macrophage HIF is sufficient to induce cell-autonomous Pdgfb expression, distal muscularization, PH, and RVH under normoxic conditions (Figure 3). Thus, HIF induces PDGF-B in macrophages, and monocyte/macrophage HIF and PDGF-B are integral to the hypoxic response of the pulmonary vasculature. We suggest that the effects of myeloid cell HIF on pulmonary vascular remodeling and hemodynamics may be largely due to secreted PDGF-B, but other HIF-regulated factors potentially contribute as well.

Our findings demonstrate that similar to distal arteriole muscularization, lung macrophages induced accumulation of alveolar myofibroblasts in the hypoxic lung (Figure 1), and myeloid-derived *Pdgfb*, *Hif1a*, and *Hif2a* were critical for this process (Figures 2, 4, and 5). Lung myofibroblasts play a key role in alveolar septal formation during normal alveologenesis in early postnatal mice, and subsequently, in the adult lung, these cells are very rare (8). In fibrotic disease, myofibroblasts are implicated in generating much of the excess extracellular matrix, and macrophages secrete profibrotic factors that recruit and activate myofibroblasts (47). In contrast, the role of monocytes/macrophages in regulating hypoxia-induced alveolar myofibroblasts has not been previously reported. We recently observed that PDGFR-β^+^ cells give rise to over 40% of hypoxia-induced myofibroblasts in the lung (R. Chandran, I. Kabir, A. Sheikh, ELH, and DMG, unpublished data) whereas SMA^+^ cells are the source of only approximately 20% (8). These results are in line with other studies suggesting that lung pericytes, which are PDGFR-β^+^SMA^–^, are an important cell type in PH (8, 48).

Approximately 10%–15% of patients with SSc develop PAH, and PAH is the leading cause of mortality in these patients. Indeed, the 3-year survival is estimated at only 49% for SSc-PAH in comparison with 84% for IPAH patients (6). One factor contributing to this heightened lethality is the muted response to standard anti-PAH treatments in SSc-PAH compared with IPAH patients (49). In addition, anti–PDGFR-β immunohistochemical staining is enhanced in the small vessels of patients with SSc-PAH in comparison with those with IPAH (50). The number of circulating monocytes does not differ between these PAH patient populations (16); however, our results indicate that in macrophages derived from these monocytes, in comparison with control humans, Pdgfb levels were more enhanced in SSc-PAH than in IPAH patients (Figure 6). Additionally, we found that macrophages from these 2 classes of PAH patients induced SMC proliferation and migration in a largely PDGF-B–dependent manner. Interestingly, a study published 25 years ago reported that PDGF-B protein level is increased in the BALF of general SSc patients (i.e., patients not evaluated for PH) compared with that of controls (51). Thus, a strategy targeting macrophage-derived PDGF-B may have efficacy in PAH.

Imatinib is a tyrosine kinase inhibitor with activity against BCR-ABL, c-KIT, and PDGFR-α and -β with applications in cancers. Daily injections of imatinib reverse pulmonary vascular remodeling, PH, and RVH due to monocrotaline in rats or chronic hypoxia in mice (52). Unfortunately, these positive results did not extrapolate to patients with PAH in the Imatinib in Pulmonary Arterial Hypertension, a Randomized Efficacy Study (IMPRES) (53). Overall, 94% of patients discontinued this oral imatinib study, and serious and unexpected adverse effects were common, including subdural hematoma. Notably, however, patients in IMPRES who were able to remain on imatinib for a long duration showed improved functional class and 6-minute walk distance. These results further emphasize the need for anti-PH therapy that targets a specific pathway (e.g., PDGF-B mediated) in a specific cell type (e.g., macrophages) in the lung.

Herein, we demonstrate that orotracheally administered PPMS polymer-formulated nanoparticles loaded with siRNA targeting *Pdgfb* substantially downregulate macrophage-derived Pdgfb, preventing hypoxia-induced distal pulmonary arteriole muscularization, PH, and RVH (Figure 7). These nanoparticles are specifically and broadly phagocytosed by lung macrophages. Previous studies have shown that intratracheal or intravenous delivery of nanoparticles carrying agents with efficacy in human PAH, including prostacyclin analogs and sildanefil, attenuates PH in experimental rodent models (54–56). To the best of our knowledge, the only prior report of nanoparticle-mediated RNA interference in this context demonstrated that intravenous delivery of antisense oligonucleotide microRNA (antimiR)-145, which aims to directly target SMCs, mitigates hypoxia/Sugen 5416-induced PH in rats; yet, in addition to the lung, this antimiR accumulates in the liver, spleen and kidney (57). The approach herein of orotracheally administering nanoparticle-loaded siRNA is advantageous as it specifically and potently targets a select gene product in lung macrophages and, thereby, promises to limit untoward effects. Furthermore, PPMS polymer-formulated nanoparticles are nontoxic and biodegradable and protect their cargo from degradation (58). However, in contrast to the prevention studies, treatment with siPdgfb nanoparticles did not reverse well established pulmonary vascular remodeling and the associated hemodynamic perturbations (Supplemental Figure 13). Future studies should assess whether initiating these nanoparticles in early stages of PH may be beneficial and whether nanoparticles that are smaller and/or specifically engineered for dual targeting of lung ECs (a major PDGF-B source) and macrophages are viable treatments for advanced disease.

A limitation of this study is the predominant use of the murine hypoxia PH model. Indeed, the main cause of death in human PAH is RV failure, and mice exposed to hypoxia develop distal pulmonary arteriole muscularization, PH, and RVH without overt RV failure. Yet, a recent echocardiographic study indicates that tricuspid annular plane systolic excursion, a measure of longitudinal RV systolic function that predicts survival in human PAH, is reduced in hypoxic mice (59, 60). Herein, for treatment groups (i.e., clodronate or siPdgfb nanoparticles or LysM-Cre–mediated deletion of *Pdgfb*, *Hif1a*, or *Hif2a*), the increase in Fulton index with 21 days of hypoxia (vs. normoxia) was on average 47% ± 12% of this increase for control groups (i.e., a ~50% reduction). Considering only the 21-day hypoxia groups and not the normoxia groups, treatments (with clodronate or siPdgfb nanoparticles or deletion of *Pdgfb*, *Hif1a*, or *Hif2a* with LysM-Cre and/or Csf1r-Mer-iCre-Mer) reduced the Fulton index by an average of approximately 25%. Importantly, in the combined hypoxia/Sugen 5416 model of severe pulmonary vascular disease, siPdgfb nanoparticles reduce distal muscularization and RVH (Supplemental Figure 14).

Taken together, our studies with an experimental model as well as cells isolated from human PAH patients demonstrate that HIFs regulate expression of PDGF-B by lung macrophages and that myeloid cell HIFs and PDGF-B play major roles in SMC remodeling, PH, and RVH. Furthermore, nanoparticle-mediated silencing of Pdgfb in lung macrophages is a preventive strategy, and further studies involving nanoparticle-mediated siRNA delivery are warranted to investigate therapies for this devastating disease.

## Methods

Further information can be found in Supplemental Methods.

### Animal studies.

Mice were obtained from The Jackson Laboratory. C57BL/6 mice were used for WT studies, and mice carrying *LysM-Cre* (32), *Csf1r-Mer-iCre-Mer* (25), *ROSA26R^mTmG/mTmG^* (61), *Pdgfb^fl/fl^* (62), *Vhl^fl/fl^* (36), *Hif1a^fl/fl^* (63) or *Hif2a^fl/fl^* (64) were previously described. Male and female mice aged 10–16 weeks and sex- and age-matched controls were used. For inducible recombination, *Csf1r-Mer-iCre-Mer* mice were injected intraperitoneally with tamoxifen (1 mg/d for 15 days), rested for 5 days, and then exposed to hypoxia.

### Hypoxia exposure and hemodynamic measurements.

Mice were placed for up to 42 days in a hypoxia (10% FiO_2_) chamber equipped with a controller and oxygen sensor (BioSpherix). In select mice, Sugen 5416 (S8442, MilliporeSigma) was injected subcutaneously (20 mg/kg) on a weekly basis during 21 days of hypoxia. Following hypoxia treatment, RVSP was measured (8). Mice were then euthanized by isoflurane inhalation, and in addition to lung harvesting, hearts were collected to determine the Fulton index, which is the weight ratio of the RV to the sum of the LV and septum (8). The technician conducting hemodynamic measurements was blinded as to treatment group and genotype of mice.

### BALF and lung harvesting.

Following euthanasia, PBS was perfused through the RV into the lungs. When the whole lung was analyzed, both the right and left lungs were harvested directly after perfusion. For BALF collection, 1 mL PBS was injected through the trachea into alveoli and then aspirated from the trachea. This procedure was repeated once, and the collected BALF was pooled. The BALF was centrifuged at 830*g* (GS-6R centrifuge, Beckman Coulter) for 10 minutes at 4°C, and the cell pellet and supernatant were collected and stored at –80°C. For FACS experiments on the residual lung, following BALF removal, the right main stem bronchus was ligated, and the right lung was removed. For immunohistochemistry, the left lung was inflated with 2% low‑melt agarose and placed in ice‑cold PBS. When the agarose solidified, the left lung was immersed in Dent’s fixative (4:1 methanol/DMSO) at 4°C overnight and the next day was washed and stored in 100% methanol at –80°C.

### Nanoparticle formulation and administration.

Nanoparticles were administered to WT mice through orotracheal instillation with minor modifications of a previously described approach (65). Briefly, mice were anesthetized by isoflurane inhalation and positioned upright hanging from their teeth with the tongue pulled forward to uncover the top of the trachea. A pipette tip was inserted orotracheally, and a maximum volume of 50 μL was instilled. Clodronate- or *Pdgfb* siRNA–loaded nanoparticles were administered twice per week starting at the onset of hypoxia and continuing for up 21 days of hypoxia. Mice receiving nanoparticles loaded with the dye DiD were maintained in normoxia for 6 hours and then euthanized. For phagocyte depletion, 50 μL of liposomes loaded with 0.25 mg clodronate or PBS and dissolved in PBS (Liposoma Research) were injected.

For nanoparticle uptake assessment or Pdgfb knockdown, PACE nanoparticles composed of acid-ended poly(pentadecalactone-co-n-methyldiethanolamineco-sebacate) with 50% lactone (PPMS-50COOH) were formulated using a modified single emulsion or double emulsion solvent evaporation technique as previously described (58). Briefly, in formulation of dye-loaded nanoparticles (~200 or ~400 nm in diameter), 0.2 wt% of DiD (Thermo Fisher Scientific) to polymer was used. DMSO (10 μL of 10 mg/mL solution) was dissolved into 50 mg of polymer immediately prior to single emulsion formulation. For *Pdgfb* siRNA and Scr RNA-loaded nanoparticles, the nucleic acid cargo (Dharmacon, 50 nM) was dissolved in sodium acetate buffer (25 mM, pH 5.8) before proceeding to the double emulsion method. Parameters of nanoparticles (stratified by siPdgfb or Scr loading) were assayed, including hydrodynamic diameter (404 ± 8 or 386 ± 7 nm), size distribution (PDI; 0.218 ± 0.004 or 0.238 ± 0.007) and zeta potential (9.4 ± 0.3 or 10.8 ± 0.5 mV) using dynamic light scattering (Zetasizer Pro, Malvern Panalytical) and siRNA loading efficiency (69.6 ± 1.2 or 64.3 ± 0.5%) using QuantIT RiboGreen assay (Thermo Fisher Scientific). Nanoparticles (0.2 mg) were suspended in 50 μL PBS and administered to mice. To confirm uptake of nanoparticles by macrophages in culture, BALF cell pellet was resuspended in murine cell culture medium (RPMI [Thermo Fisher Scientific], 10% fetal bovine serum [FBS; Invitrogen, Thermo Fisher Scientific], 5% penicillin/streptomycin [Life Technologies, Thermo Fisher Scientific]) and incubated with 0.25 mg/mL DiD-loaded nanoparticles for 6 hours at 37°C.

### Vibratome section preparation and immunohistochemistry.

For immunohistochemical analysis, left lungs stored in 100% methanol were subjected to peroxidase deactivation by incubation in 5% H_2_O_2_/methanol for 15 minutes at room temperature and then sequentially rehydrated in 75%, 50%, and 25% and 0% methanol in PBS. A vibratome was used to cut the rehydrated lung into 150 μm thick sections, which were incubated in IHC blocking buffer (5% goat serum in 0.5% Triton X-100/PBS [PBS‑T]) at 4°C overnight and then stained with primary antibodies in IHC blocking buffer for 3 days at 4°C. Subsequently, sections were washed 3 times in PBS‑T, incubated in secondary antibodies in IHC blocking buffer overnight at 4°C, washed 5 times in PBS‑T, mounted on slides with Dako mounting medium, and stored at 4°C. Primary antibodies used were rat anti–MECA-32 (1:15, Developmental Studies Hybridoma Bank), rat anti–CD31-FITC clone MEC13.3 (1:250, 561813, BD Biosciences), mouse anti–CD64-APC clone X54-5/7.1 (1:250, 139306, Biolegend), rat anti–CD68-APC clone FA-11 (1:50, 130-102-585, Miltenyi Biotec), rat anti-aquaporin1 (1:100, ab15080, Abcam), and mouse anti–SMA‑Cy3 clone 1A4 (1:250, A2547, MilliporeSigma). Secondary antibody used was Alexa Fluor 488 anti-rat (1:250, catalog A-11006, Invitrogen, Thermo Fisher Scientific). Nuclei were stained with DAPI (1:500).

### Imaging.

Images of the stained sections were acquired using confocal microscopes (PerkinElmer UltraView VOX spinning disc or Leica SP8 point scanning) or an upright microscope (Nikon ECLIPSE 80i). Adobe Photoshop was used to process images. For analysis of distal muscularization, we focused on 2 specific arteriole beds in the left lung previously described and denoted as L.L1.A1.L1 and L.L1.A1.M1 (8, 9). Their nomenclature derives from the nearest airways that have a stereotyped branching pattern in the adult mouse (8, 66). Based on their diameter and branching pattern, pulmonary arterioles are classified as proximal (P; >75 mm diameter), middle (M; 25 to 75 mm), and distal (D; <25 mm) and the names L, left main bronchus; L1, L2, L3, lateral branches; M1, M2 medial branches; A1, A2 anterior branches (8).

### Human monocyte isolation and differentiation to macrophages.

Fresh whole blood from IPAH and SSc-PAH patients of the Pulmonary Vascular Disease clinic at Yale University School of Medicine and healthy controls were provided to the Greif lab as deidentified samples. Monocytes were isolated and differentiated into macrophages based on methods described previously (37, 38). In brief, fresh whole blood was diluted 3-fold in HBSS, loaded on a Ficoll-Histopaque column (Thermo Fisher Scientific), and centrifuged for 30 minutes at 830*g*. The PBMC phase was aspirated, diluted 3-fold in HBSS, and centrifuged for 10 minutes at 830*g*. To ensure platelet removal, the pellet was resuspended in 3 mL HBSS and centrifuged for an additional 10 minutes at 830*g*. The pellet was then resuspended in RPMI with 10% FBS, and cells were allowed to adhere to a plastic cell culture dish for 1 hour at 37°C. Monocytes preferentially adhere to plastic (37) (Supplemental Figure 9, A and B). Floating cells were discarded, and adherent cells were washed with PBS and either incubated with 5 mM EDTA in PBS for 10 minutes and collected for staining and flow cytometry or cultured in macrophage differentiation medium (ImmunoCult-SF macrophage medium and 1 ng/mL macrophage colony-stimulating factor, both from StemCell Technologies). The medium was replaced by fresh macrophage differentiation medium on the fourth day. On day 6, the medium was changed to ImmunoCult-SF macrophage medium, and 12 hours later, conditioned medium was collected and cells harvested. For hypoxia studies, macrophages derived from monocytes of healthy donors were exposed to either normoxia or 3% O_2_ for 12 hours in RPMI supplemented with 1% FBS and 5% penicillin-streptomycin.

### hPASMC culture and proliferation assay.

hPASMCs (American Type Culture Collection) were cultured up to passage 6 in M199 medium supplemented with 10% FBS, 1% penicillin/streptomycin, 2 ng/mL fibroblast growth factor (Promega), and 3 ng/mL epidermal growth factor (Promega). Proliferation was assessed as previously described with minor modifications (67). hPASMCs were trypsinized and cultured overnight on culture slides (BD Falcon) precoated with fibronectin (10 μg/mL in PBS). On the next day, the cells were washed with PBS and serum starved overnight in M199 supplemented with 0.5% FBS. Cells were then washed in PBS and cultured for 24 hours in medium conditioned by human control or patient-derived macrophages that had or had not been pretreated with 20 μg/mL IgG control or anti–PDGF-B blocking antibody (R&D Systems, Bio-Techne) for 1 hour at 37°C. For the final 10 hours of this incubation, 10 μg/mL BrdU (MilliporeSigma) was added to the cells. Slides were fixed in 4% paraformaldehyde for 30 minutes, rinsed in 0.3% Tris and 1.5% glycine in water for 15 minutes, incubated in 2N HCl for 30 minutes at 37°C, washed with 0.1 M boric acid, and then incubated in 1% FBS in PBS-T for 1 hour. hPASMCs were stained with rat anti-BrdU primary antibody (1:100, Bio-Rad catalog MCA2060) in 1% FBS in PBS-T for 1 hour, washed 3 times in 0.5% Tween 20 in PBS, and then incubated with goat anti-rat secondary antibody conjugated to Alexa Fluor 488 (1:500, Molecular Probes catalog A-11006) and PI (1:500, MilliporeSigma) in 1% FBS in PBS-T for 1 hour. Finally, slides were washed 3 times in 0.5% Tween 20 in PBS and mounted on slides using fluorescence mounting medium (Dako). Proliferation was calculated as the percentage of total PI^+^ hPASMCs that were BrdU^+^. For each control or patient, at least 10 fields of view were scored.

### SMC migration assay.

Cell migration was assessed in a similar manner as we previously described (67). Briefly, hPASMCs were trypsinized and immediately added to the top of Boyden chamber polycarbonate membranes (Corning Costar, 8 μm pores). The lower compartment of the Boyden chamber contained medium conditioned by human control and patient-derived macrophages that was or was not pretreated with 20 μg/mL anti–PDGF-B blocking antibody or IgG control for 1 hour at 37°C. hPASMCs were allowed to migrate for 8 hours toward the lower chamber at which time the membrane was fixed in 4% paraformaldehyde for 30 minutes, stained with 0.1% Crystal Violet, and washed with water. The upper surface of the membrane was scraped with a cotton swab to remove nonmigrated cells, and cells on the bottom surface (i.e., migrated cells) were imaged and counted.

### Statistics.

All data are presented as mean values ± standard deviation. Student’s *t* test (unpaired, 2 tailed) and 1-way ANOVA were used to compare means of 2 groups and multiple groups, respectively (GraphPad Prism software). The statistical significance threshold was set at *P* ≤ 0.05. All tests assumed normal distribution.

### Study approval.

All procedures involving human subjects were approved by the Institutional Review Board of Yale University (IRB 1307012431 and 1005006865), and we complied with all relevant ethical regulations. Written informed consent was obtained from all participants prior to inclusion in the study. All mouse experiments were approved by the Institutional Animal Care and Use Committee at Yale University.

## Author contributions

AN, JMD, ACK, MS, WMS, and DMG conceived of and designed experiments, and AN, JMD, and ACK performed them. IS provided patient samples, CR helped with patient clinical data, and JH and ELH provided infrastructure for the human studies. AN and DMG analyzed the results. AN and DMG prepared the figures, and AN, ACK, and DMG wrote the manuscript. All authors reviewed and provided input on the manuscript.

## Supplementary Material

Supplemental data

## Version 1 02/16/2021

In-Press Preview

## Version 2 03/22/2021

Electronic publication
