# Supplementary material for: Macrophage-derived PDGF-B induces muscularization in murine and human pulmonary hypertension
Source: JCI Insight. 2021 Mar 22;6(6):e139067. doi: 10.1172/jci.insight.139067 (PMC8026182; doi:10.1172/jci.insight.139067)
Supplement: Supplemental data [file jciinsight-6-139067-s144.pdf]

SUPPLEMENTARY INFORMATION

Macrophage-derived PDGF-B induces muscularization in murine and human pulmonary hypertension

Aglaia Ntokou, Jui M. Dave, Amy C. Kauffman, Maor Sauler, Changwan Ryu, John Hwa, Erica L. Herzog, Inderjit Singh, W. Mark Saltzman and Daniel M. Greif

List of Supplementary Information

1. Supplementary Methods
2. Supplementary Reference
3. Supplementary Figures S1-S14
4. Supplementary Figure Legends
5. Supplementary Tables

Supplementary Methods

Respiratory mechanics

Mice were mechanically ventilated as previously described (1). Briefly, mice were injected intraperitoneally with urethane (0.1 mg/kg; Sigma), and the trachea was cannulated. Paralysis was achieved with intraperitoneal administration of pancuronium bromide (0.1 mg/kg; Sigma). Mice were then connected to a ventilator (flexiVent, SCIREQ) and ventilated at 10 cc/kg. Pressure-volume curves and lung compliance was assessed using flexiWare software (SCIREQ). Three measurements of each parameter were measured and averaged.

Flow cytometry

Harvested right lungs from mice were placed in 1 ml dispase (1 U/ml, StemCell Technologies) in a cell culture dish on ice. The tissue was minced into small pieces and then digested in 5 ml dispase containing 1% penicillin/streptomycin and 2.1% DNase (Serva) for 1 h at 37°C. The digested tissue was sequentially passed through 100-µm and 40-µm meshes, and the filtrate was centrifuged at 830*g* (GS-6R centrifuge, Beckman Coulter) for 10 min at 4°C. This cell pellet or the BALF cell pellet was resuspended in flow cytometry staining buffer (Thermo Fisher Scientific, eBioscience). These cells or isolated human monocytes were stained for 30 min at 4°C with antibodies raised in mice and diluted in flow cytometry staining buffer (1:100 or 1:250, respectively). All antibodies were purchased from Biolegend except for anti-CD31-FITC clone MEC 13.3 (cat #561813, BD Biosciences), which was used for murine samples. Additional antibodies used for murine samples were anti-CD64-APC clone X54-5/7.1 (cat #139306), anti-Ly6G-FITC clone 1A8 (cat #127605), anti-CD31-Pacific Blue clone 390 (cat #102421), anti-CD326-FITC clone G8.8 (cat #118207), and/or CD45-PE clone 30-F11 (cat

#103106). For human samples, anti-CD3-PE clone HIT3a (cat #300307), anti-CD14-FITC clone HCD14 (cat #325603) and anti-CD19-APC clone SJ25C1 (cat #363005). The stained samples were then washed in flow cytometry staining buffer and centrifuged at 532*g* for 3 min at 4°C, and the pellet was resuspended and fixed in flow cytometry buffer containing 0.15% paraformaldehyde (Sigma-Aldrich) on ice for 15 min. The fixed cells were filtered using a 100 µm filter and centrifuged at 532*g* for 10 min at 4°C. Finally, samples were resuspended in 200 µl flow cytometry buffer and stored at 4°C prior to flow cytometric analysis or sorting with a FACSAria II cytometer (BD Biosciences). For isolation of GFP^{+} cells from *LysM-Cre, ROSA26R*$^{(mTmG/mTmG)}$ mice, a single cell suspension of lungs was directly subjected to FACS without staining or fixation. For each fluorophore, the excitation wavelength and bandpass filters were: APC 640 nm and 670/30 nm; Pacific Blue 405 nm 450/50 nm; PE 488 nm and 582/15 nm; FITC or GFP 488 nm and 525/50 nm.

Cryosection preparation and immunostaining

The lungs, heart and liver were harvested from mice, and lungs were inflated with 1:1 PBS:optical cutting temperature compound (OCT; Tissue Tek). These organs were fixed in 4% paraformaldehyde overnight at 4°C. Fixed organs were washed in PBS, cryoprotected overnight in 30% sucrose at 4°C, fast frozen in OCT and stored as cryoblocks at -80°C. Prior to staining, 10 µm thick sections were cut from cryoblocks, placed on slides to dry and stored at -20°C. Slides were thawed at RT, fixed in acetone at -20°C for 20 min, blocked in IHC blocking buffer for 1 h at RT and incubated with anti-CD68-APC clone FA-11 (1:50, cat #130-102-585, Miltenyi Biotec) for 3 h. Slides were then washed with PBS, stained with DAPI for 20 min and mounted with Dako mounting medium (Agilent).

Paraffin section preparation and hematoxylin and eosin staining

The lung, liver and heart were harvested from mice and fixed in 4% paraformaldehyde overnight at 4°C. Fixed organs were washed in PBS, dehydrated and embedded in paraffin blocks. Sections were cut at 3 μm thickness. Slides were deparaffinized by incubating at 59°C for 1 h, rehydrated, stained with hematoxylin and eosin (H&E) per standard protocols and mounted with Pertex (Vectashield).

Culture of isolated murine cells

Lung GFP^+ cells isolated by FACS from *LysM-Cre*, *ROSA26R$^{(mTmG/mTmG)}$* mice or BALF cells from wild type mice were cultured in murine cell culture medium (RPMI, 10% FBS, 5% penicillin/streptomycin). Cells were allowed to adhere to a plastic dish for 1 h, washed in PBS and then either exposed to normoxia or 3% O_2 for 6 h in murine cell culture medium or in select experiments, BALF cells were subjected to Pdgfb knockdown.

siRNA-mediated knockdown of Pdgfb

BALF cell pellet, which predominately consists of $CD64^+Ly6G^-$ macrophages (see Fig. 1B), was collected from mice and resuspended in RPMI, 1% FBS, 5% penicillin/streptomycin for 1 h. The cells were then transfected with Lipofectamine 2000 (Life Technologies) containing 50 μM siRNA targeted against Pdgfb or Scr RNA (Dharmacon) for 6 h. Cells were washed in PBS, cultured for 72 h in murine cell culture medium and subjected to quantitative real-time (qRT)-PCR analysis.

Quantitative real-time PCR analysis

In murine studies, the RNeasy Mini Kit (Qiagen) was used to isolate RNA from BALF cells or FACS-sorted $CD64^{+}Lys6G^{-}$ cells from either the whole lung, BALF or residual right lung after BALF collection. For human studies, RNA was isolated from macrophages with the PureLink^{TM} RNA Minikit (Invitrogen). RNA was reverse transcribed with the iScript cDNA Synthesis Kit (Biorad). The expression levels of selected genes were determined by qRT-PCR and normalized to Gapdh. Primer sequences are listed in Table S1.

Enzyme-linked immunosorbent assay

PDGF-B protein levels in mouse BALF and medium conditioned by human macrophages were measured by enzyme-linked immunosorbent assay (ELISA) using Thermo Fisher Scientific kits (cat #EM63RB and #BMS2071, respectively). Briefly, frozen cell-free mouse BALF and human macrophage conditioned medium were thawed at RT and diluted in assay diluent. Antigen binding, incubation with anti-PDGF-B biotin conjugate and streptavidin-horseradish peroxidase, reaction with tetramethyl-benzidine substrate and detection of product was carried out per the manufacturer's instructions.

Western blot

The BALF cell pellet was lysed in 1.5X Laemmli sample buffer at 95°C for 10 min. Protein samples were resolved by 10% SDS-PAGE and transferred to Immobilon PVDF membranes (Millipore). Membranes were blocked with 5% nonfat dry milk, washed in tris-buffered saline with 0.1% Tween 20 (TBS-Tween) and then probed with primary antibodies overnight at 4°C. On the next day, membranes were washed with TBS-Tween, incubated with

HRP-conjugated secondary antibodies (Dako) for 1 h, washed with TBS-Tween, developed with Supersignal West Femto Maximum Sensitivity Substrate (Pierce) and analyzed with the G:BOX imaging system (Syngene). Primary antibodies used for Western blot analysis were raised in rabbits and were anti-HIF1-α and anti-HIF2-α (each from Novus Biologicals, 1:100) and anti-beta actin (Abcam, 1:2000).

Cryopreserved PBMCs

Blood was phlebotomized from IPAH and SSc-PAH patients or healthy controls and centrifuged. The cell pellet was diluted 1:2 (v/v) in PBS and then added to the top layer of 12 ml Histopaque and centrifuged at 2500 rpm for 25 min. PBMCs were isolated, washed three times with PBS, resuspended in 70% DMEM, 20% FBS, 10% DMSO and frozen in boxes containing isopropyl alcohol at -80°C overnight. The next day, samples were transferred to liquid nitrogen for long-term storage. When ready for use, de-identified frozen PBMCs were provided to the Greif lab and thawed at 37°C. Thawed samples were washed two times in HBSS and used as described for PBMCs isolated from fresh blood in the main Methods.

Supplementary Reference

1. Nouws J, Wan F, Finnemore E, Roque W, Kim SJ, Bazan I, et al. MicroRNA miR-24-3p reduces DNA damage responses, apoptosis, and susceptibility to chronic obstructive pulmonary disease. *JCI Insight.* 2021;6(2).

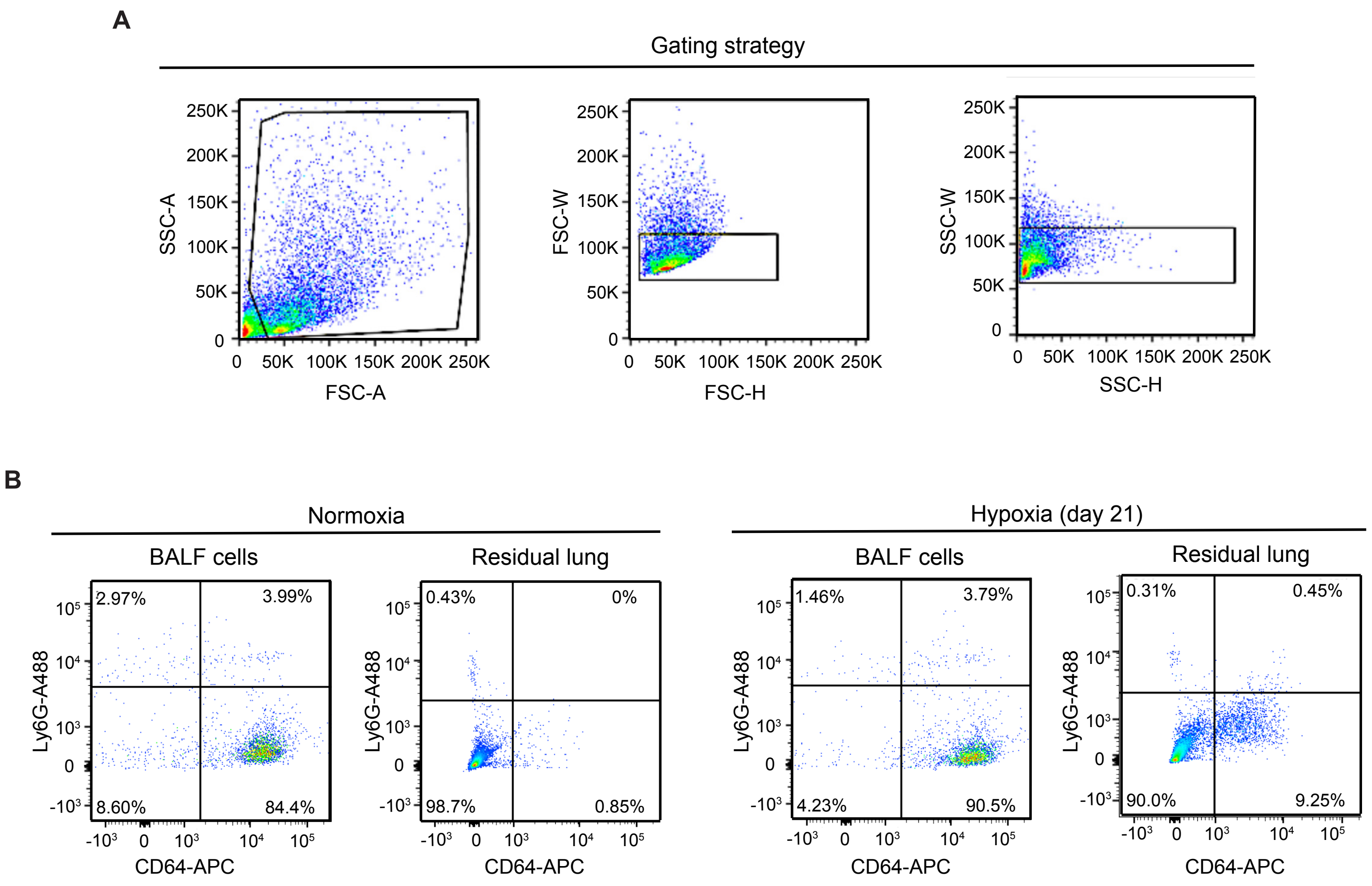
A
Gating strategy
SSC-A
FSC-A
FSC-W
FSC-H
SSC-W
SSC-H
250K
200K
150K
100K
50K
0
B
Normoxia
Hypoxia (day 21)
BALF cells
Residual lung
Ly6G-A488
CD64-APC
2.97%
3.99%
8.60%
84.4%
0.43%
0%
98.7%
0.85%
1.46%
3.79%
4.23%
90.5%
0.31%
0.45%
90.0%
9.25%

Wild type; BALF

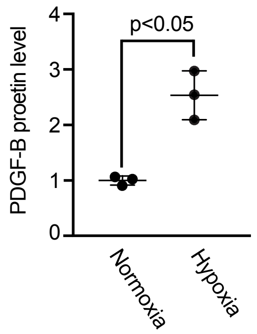

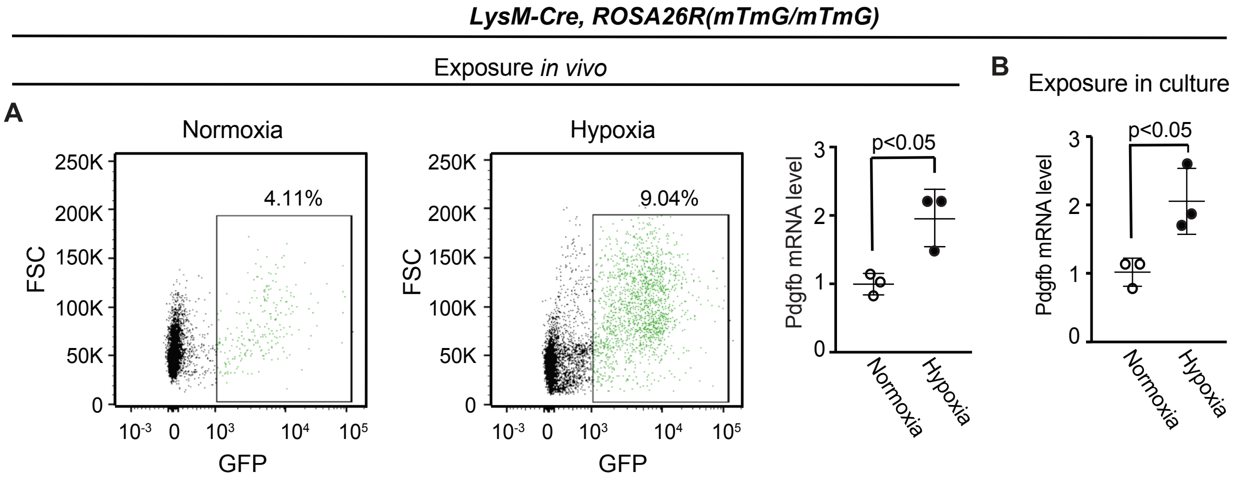

LysM-Cre, ROSA26R(mTmG/mTmG)
Exposure in vivo
A
Normoxia
Hypoxia
4.11%
9.04%
FSC
GFP
250K
200K
150K
100K
50K
0
10-3
0
10³
10⁴
10⁵
Pdgfb mRNA level
p<0.05
3
2
1
0
Normoxia
Hypoxia
B
Exposure in culture
Pdgfb mRNA level
p<0.05
Normoxia
Hypoxia

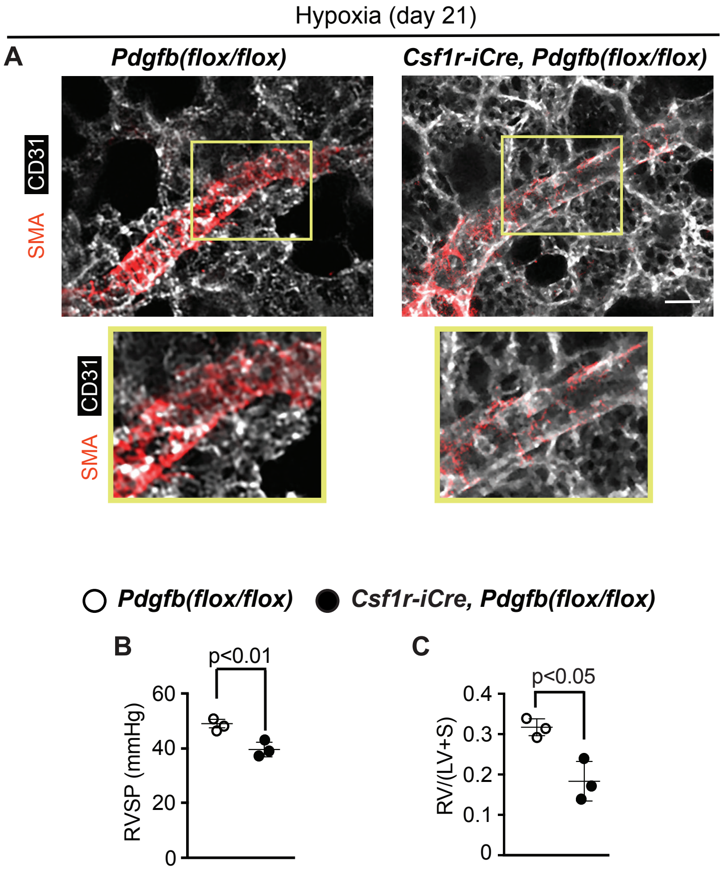
Hypoxia (day 21)
A
Pdgfb(flox/flox)
Csf1r-iCre, Pdgfb(flox/flox)
CD31
SMA
CD31
SMA
Pdgfb(flox/flox)
Csf1r-iCre, Pdgfb(flox/flox)
B
p<0.01
RVSP (mmHg)
60
40
20
0
C
p<0.05
RV/(LV+S)
0.4
0.3
0.2
0.1
0

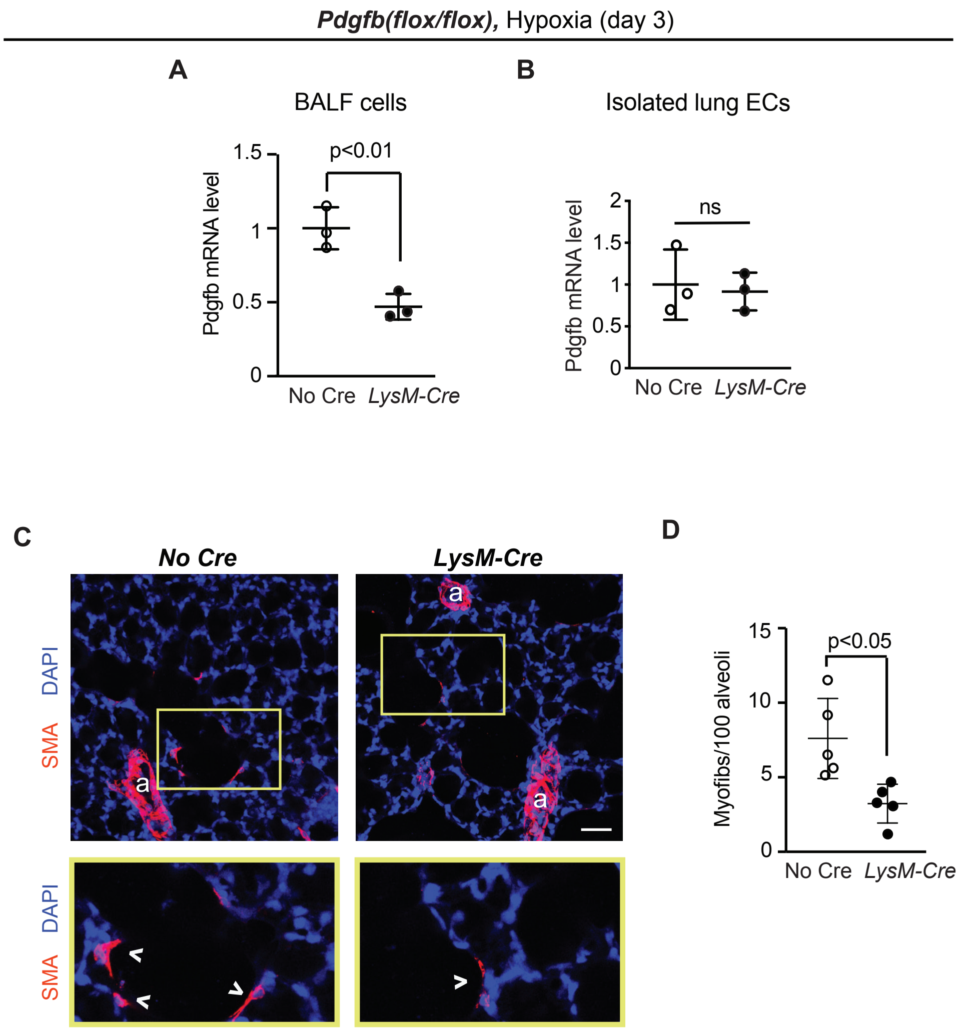
Pdgfb(flox/flox), Hypoxia (day 3)
A
BALF cells
p<0.01
Pdgfb mRNA level
1.5
1
0.5
0
No Cre
LysM-Cre
B
Isolated lung ECs
ns
Pdgfb mRNA level
2
1.5
1
0.5
0
No Cre
LysM-Cre
C
No Cre
LysM-Cre
DAPI
SMA
a
D
p<0.05
Myofibs/100 alveoli
15
10
5
0
No Cre
LysM-Cre

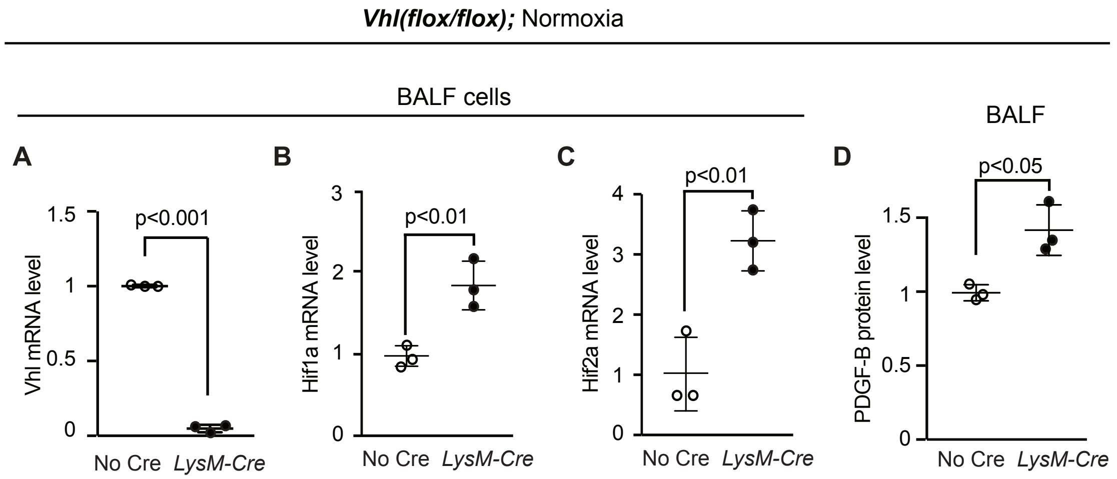
Vhl(flox/flox); Normoxia
BALF cells
BALF
A
B
C
D
Vhl mRNA level
p<0.001
1.5
1
0.5
0
No Cre
LysM-Cre
Hif1a mRNA level
p<0.01
3
2
1
0
Hif2a mRNA level
p<0.01
4
3
2
1
0
PDGF-B protein level
p<0.05
1.5
1
0.5
0

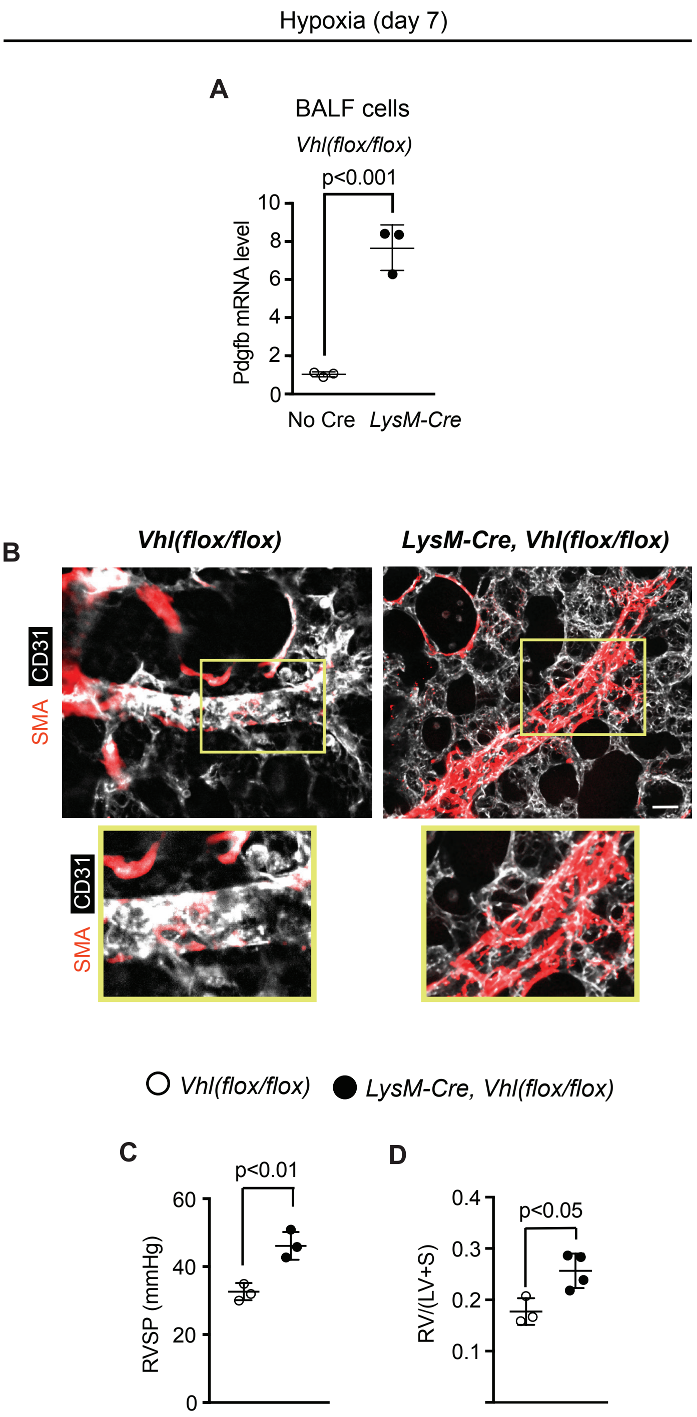

Hypoxia (day 7)
A
BALF cells
Vhl(flox/flox)
p<0.001
Pdgfb mRNA level
0
2
4
6
8
10
No Cre
LysM-Cre
B
Vhl(flox/flox)
LysM-Cre, Vhl(flox/flox)
CD31
SMA
CD31
SMA
Vhl(flox/flox)
LysM-Cre, Vhl(flox/flox)
C
p<0.01
RVSP (mmHg)
0
20
40
60
D
p<0.05
RV/(LV+S)
0.1
0.2
0.3
0.4

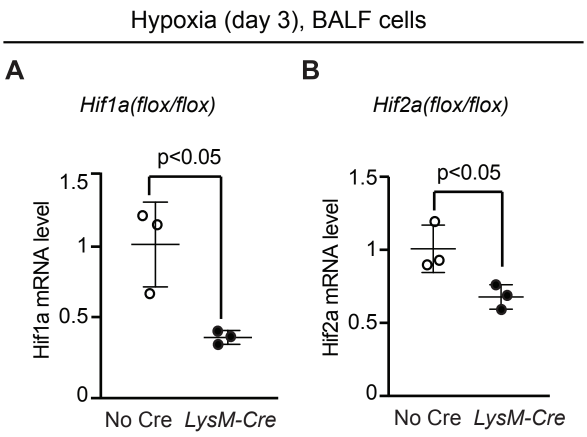
Hypoxia (day 3), BALF cells
A
Hif1a(flox/flox)
p<0.05
Hif1a mRNA level
1.5
1
0.5
0
No Cre
LysM-Cre
B
Hif2a(flox/flox)
p<0.05
Hif2a mRNA level
1.5
1
0.5
0
No Cre
LysM-Cre

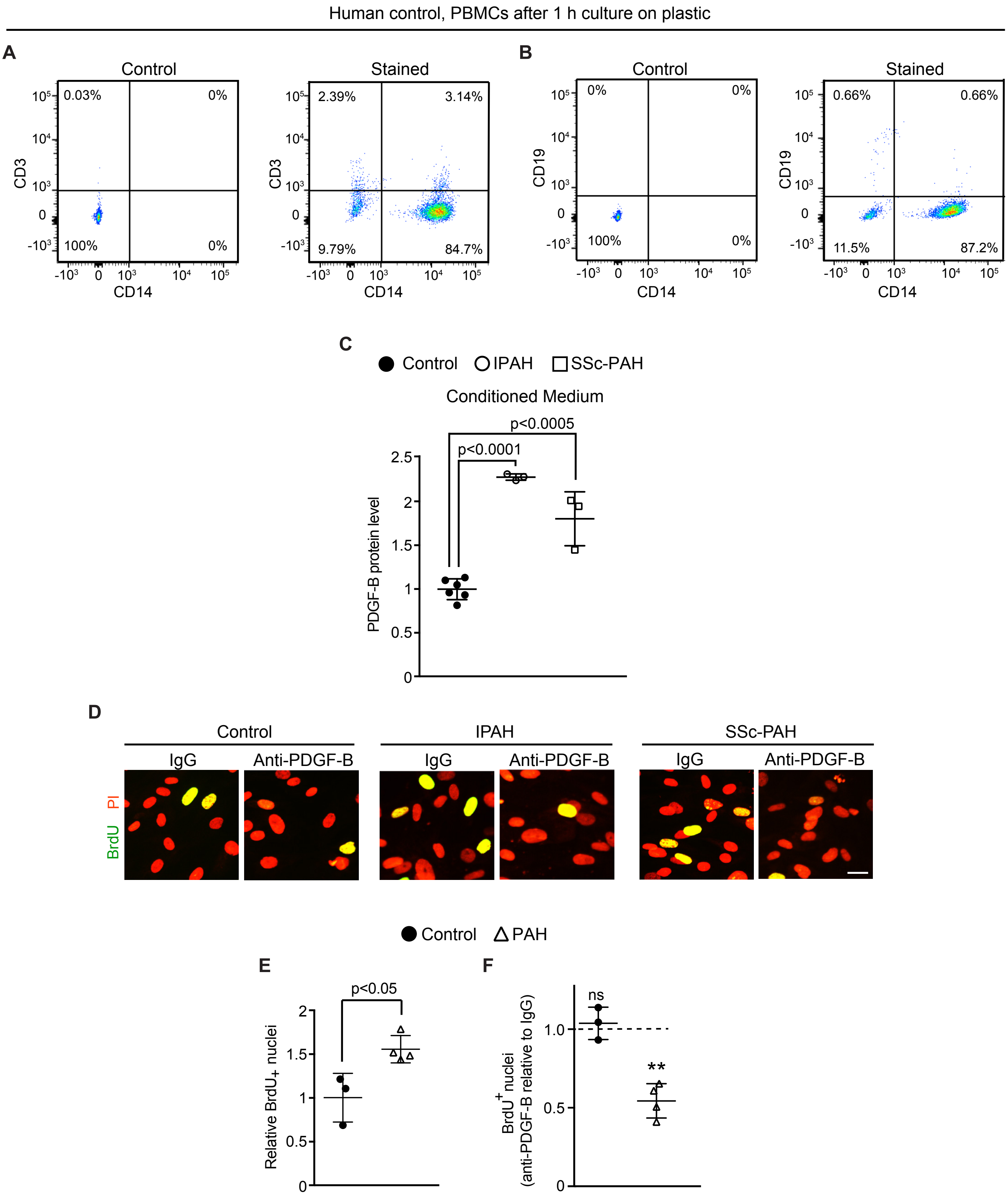
Human control, PBMCs after 1 h culture on plastic
A
Control
Stained
0.03%
0%
100%
0%
2.39%
3.14%
9.79%
84.7%
CD3
CD14
B
Control
Stained
0%
0%
100%
0%
0.66%
0.66%
11.5%
87.2%
CD19
CD14
C
Control
IPAH
SSc-PAH
Conditioned Medium
p<0.0005
p<0.0001
PDGF-B protein level
D
Control
IPAH
SSc-PAH
IgG
Anti-PDGF-B
PI
BrdU
Control
PAH
E
p<0.05
Relative BrdU+ nuclei
F
ns
**
BrdU+ nuclei
(anti-PDGF-B relative to IgG)

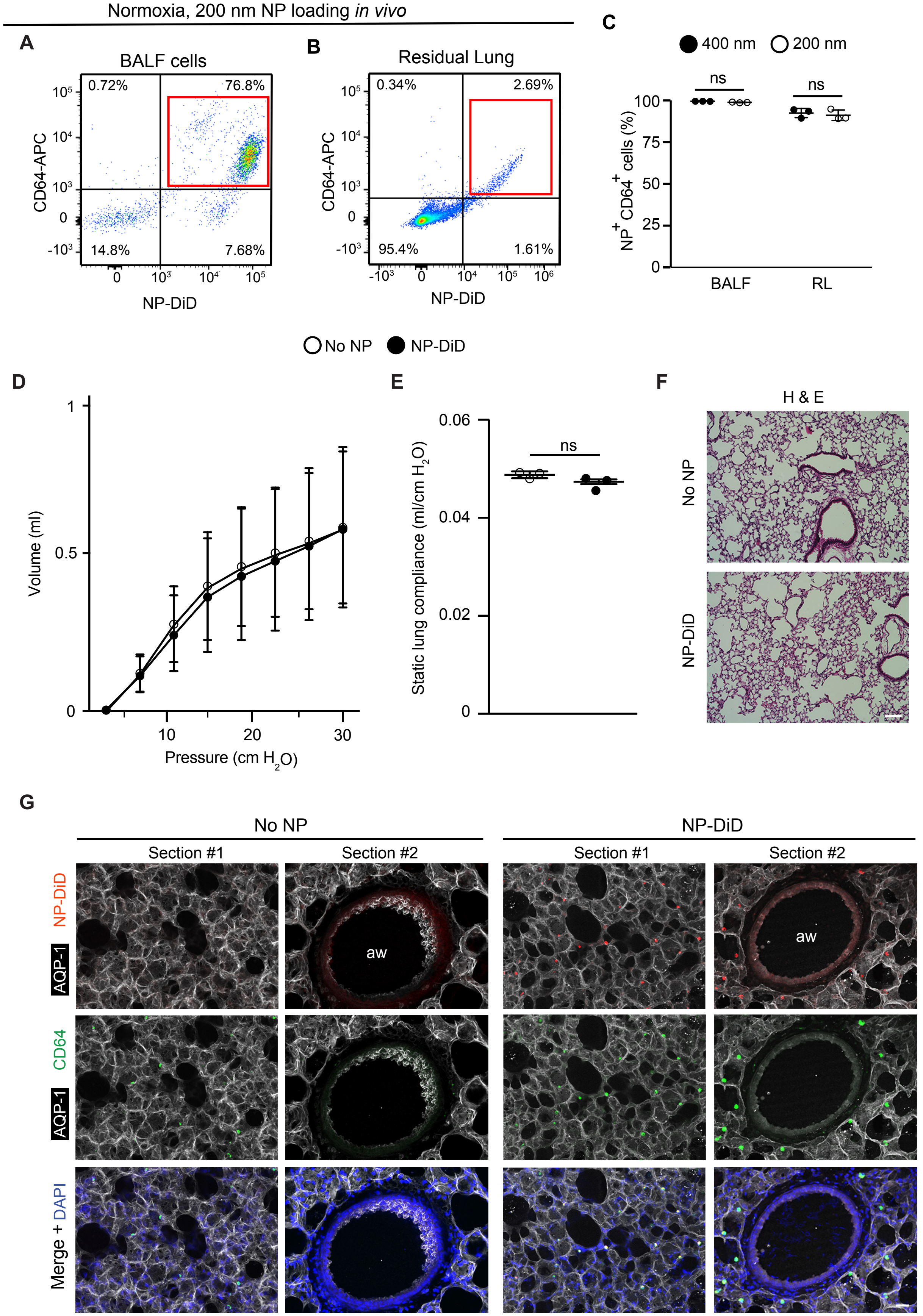

Normoxia, 200 nm NP loading in vivo
A
BALF cells
0.72%
76.8%
14.8%
7.68%
CD64-APC
NP-DiD
B
Residual Lung
0.34%
2.69%
95.4%
1.61%
CD64-APC
NP-DiD
C
400 nm
200 nm
ns
ns
NP+ CD64+ cells (%)
BALF
RL
No NP
NP-DiD
D
Volume (ml)
Pressure (cm H2O)
E
ns
Static lung compliance (ml/cm H2O)
F
H & E
No NP
NP-DiD
G
No NP
NP-DiD
Section #1
Section #2
Section #1
Section #2
NP-DiD
AQP-1
CD64
AQP-1
Merge + DAPI
aw
aw

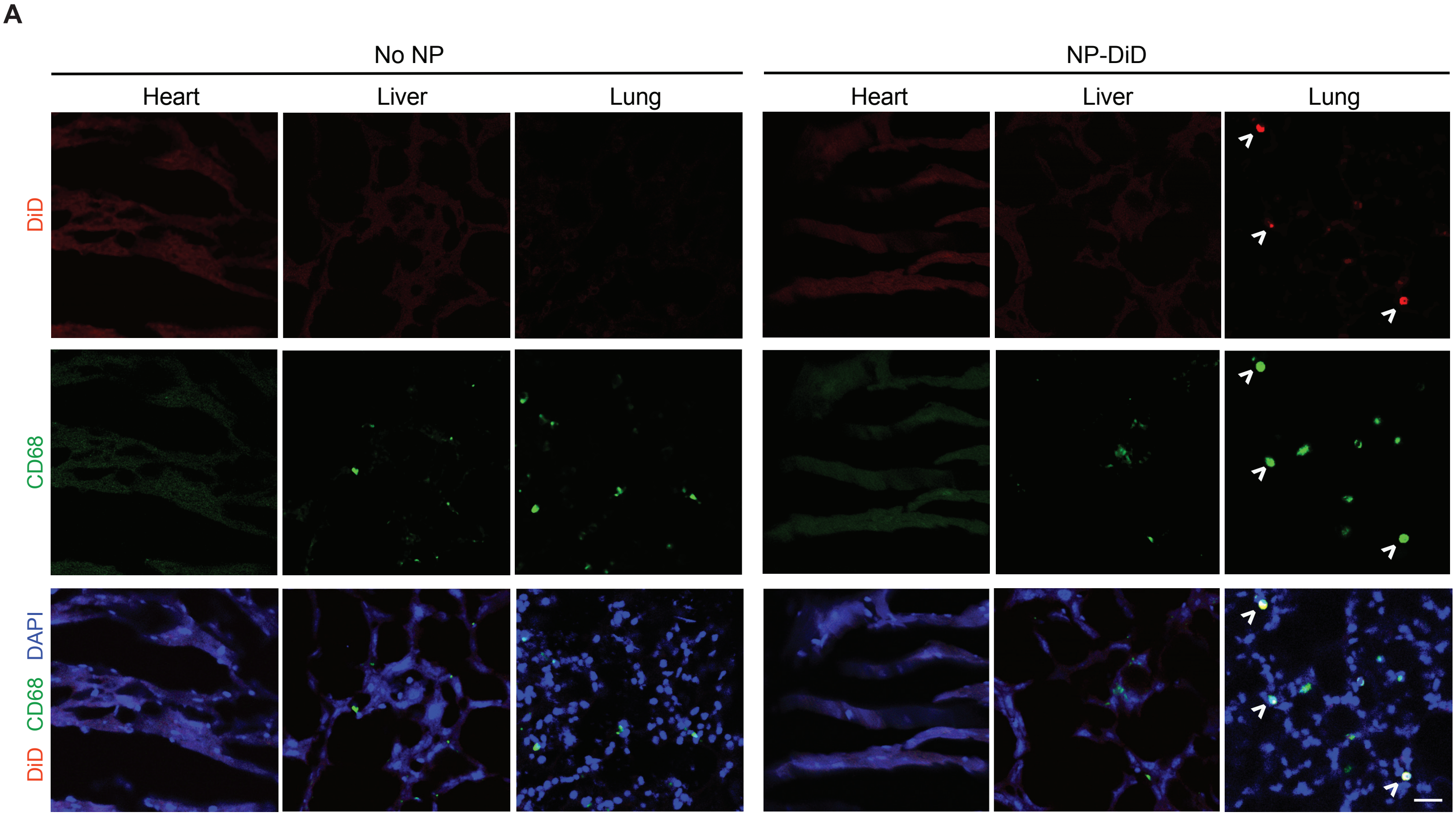
A
No NP
NP-DiD
Heart
Liver
Lung
Heart
Liver
Lung
DiD
CD68
DiD CD68 DAPI

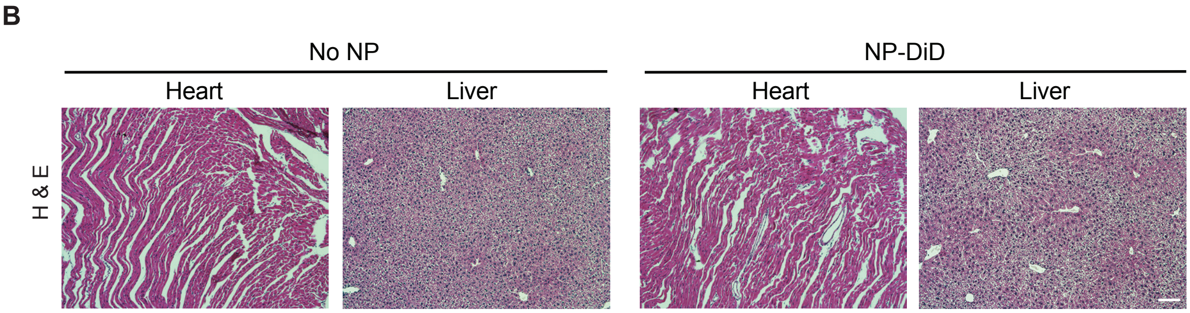
B
No NP
NP-DiD
Heart
Liver
Heart
Liver
H & E

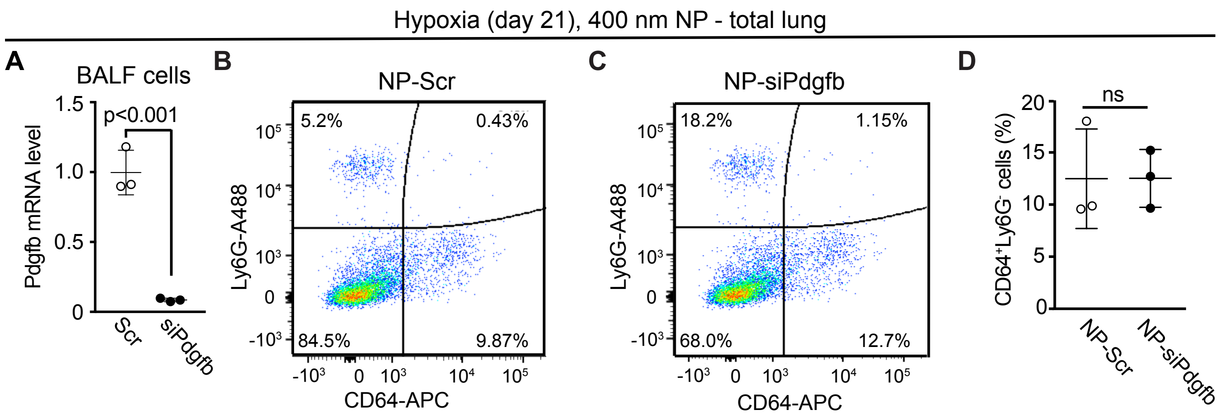
Hypoxia (day 21), 400 nm NP - total lung
A
BALF cells
p<0.001
Pdgfb mRNA level
1.5
1.0
0.5
0
Scr
siPdgfb
B
NP-Scr
5.2%
0.43%
84.5%
9.87%
Ly6G-A488
CD64-APC
C
NP-siPdgfb
18.2%
1.15%
68.0%
12.7%
Ly6G-A488
CD64-APC
D
ns
CD64+Ly6G- cells (%)
20
15
10
5
0
NP-Scr
NP-siPdgfb

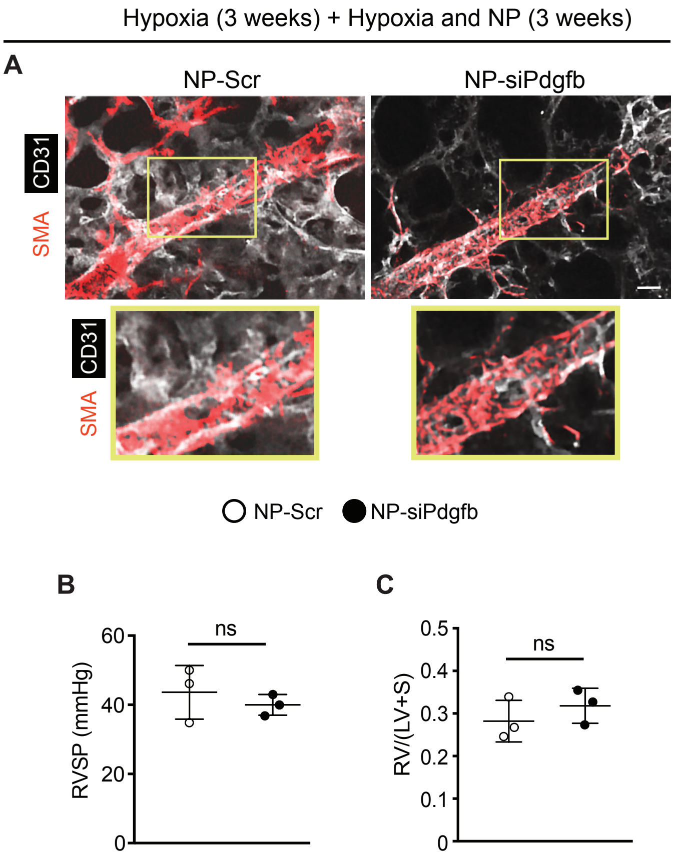
Hypoxia (3 weeks) + Hypoxia and NP (3 weeks)
A
NP-Scr
NP-siPdgfb
CD31
SMA
CD31
SMA
NP-Scr
NP-siPdgfb
B
RVSP (mmHg)
ns
60
40
20
0
C
RV/(LV+S)
ns
0.5
0.4
0.3
0.2
0.1
0

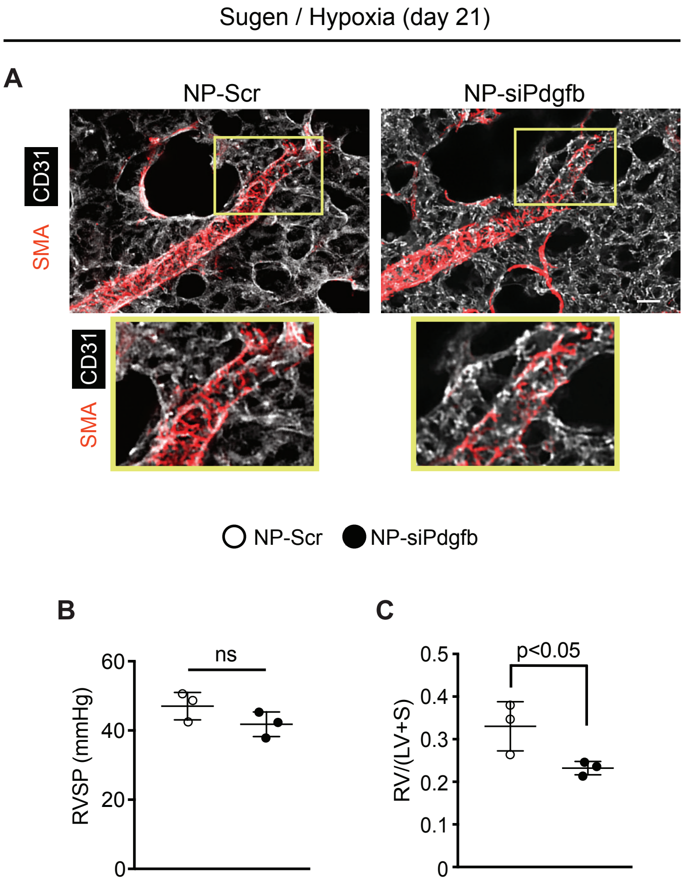
Sugen / Hypoxia (day 21)
A
NP-Scr
NP-siPdgfb
CD31
SMA
CD31
SMA
NP-Scr
NP-siPdgfb
B
ns
RVSP (mmHg)
60
40
20
0
C
p<0.05
RV/(LV+S)
0.5
0.4
0.3
0.2
0.1
0

Supplementary Figure Legends

Figure S1. Flow cytometry of cells from the mouse lung. Lung cells of wild type mice were subjected to flow cytometric analysis. **A,** For all flow cytometric experiments included throughout all figures and supplementary figures, single cell suspensions of lungs were gated to remove non-cellular particles or doublets. Forward and side scatter parameters (FSC and SSC, respectively) were used to achieve single cell populations before gating for specific markers. **B,** Dot plots of single cells from BALF and residual lung of mice maintained in normoxia (left) or exposed to 21 days of hypoxia (FiO_2 10%; right) are shown. Cells were stained for CD64 and Ly6G in order to identify $CD64^{+}Ly6G^{-}$ macrophages by flow cytometry. n=3 mice per condition.

Figure S2. Exposure of mice to hypoxia increases PDGF-B protein levels in BALF. BALF was isolated from wild type mice exposed to hypoxia (10% FiO_2) for 3 days or normoxia and then centrifuged. The cell-free supernatant was subjected to ELISA to measure PDGF-B protein, and PDGF-B protein levels relative to normoxia are shown. n=3 mice per treatment group, ELISA was done in duplicate for each mouse. Student's t-test was used.

Figure S3. Hypoxia increases Pdgfb levels in lung cells marked via LysM-Cre. GFP^{+} cells were isolated by FACS of the total lungs from *LysM-Cre, ROSA26R$^{(mTmG/mTmG)}$* mice, and Pdgfb mRNA was measured by qRT-PCR. **A,** FACS plots from mice exposed to hypoxia for 21 days or maintained in normoxia. Pdgfb mRNA levels in isolated GFP^{+} cells were measured. **B,** GFP^{+} cells were isolated from normoxic mice, exposed in culture to normoxia or 3% O_2 for 12 h and then Pdgfb mRNA level was measured. n=3 mice per treatment group and qRT-PCR was done in triplicate. Student's t-test was used.

Figure S4. ***Pdgfb*** **deletion in CSF1R$^+$ cells attenuates distal muscularization and PH.** *Pdgfb$^{(flox/flox)}$* mice also carrying no Cre or *Csf1r-Mer-iCre-Mer* were injected with tamoxifen, rested and then exposed to hypoxia for 21 days or maintained in normoxia. **A**, Lung vibratome sections with distal arterioles in the L.L1.A1 regions were stained for SMA and EC marker CD31. Boxed regions are shown below as close-ups. Scale bar, 25 μm. **B, C,** RVSP and Fulton index measurements are shown. Student's t-test was used. n=3 mice per genotype.

Figure S5. Deletion of ***Pdgfb*** **in LysM$^+$ cells reduces Pdgfb in BALF cells and lung myofibroblasts with hypoxia.** *Pdgfb$^{(flox/flox)}$* mice also carrying no Cre or *LysM-Cre* were subjected to hypoxia for 3 days. **A, B,** qRT-PCR was used to measure Pdgfb transcript levels in BALF cells and in CD31$^+$CD45$^-$ ECs isolated by FACS of the whole lung, respectively. n=3 mice for each genotype, qRT-PCR was done in triplicate. **C**, Lung vibratome sections were stained for SMA and nuclei (DAPI), and arterioles are labeled with a. Boxed regions are shown below as close-ups with arrowheads indicating alveolar myofibroblasts. **D**, The number of myofibroblasts were quantified per 100 alveoli. More than 500 alveoli were quantified per mouse. n=5 mice per gentoype. Scale bar, 25 μm. Student's t-test was used.

Figure S6. ***Vhl*** **deletion in LysM$^+$ cells induces Hifa and PDGF-B in BALF.** *LysM-Cre* mice also carrying no Cre or *Vhl$^{(flox/flox)}$* were maintained in normoxia. **A-C**, BALF was removed, and cellular levels of the indicated transcript were measured by qRT-PCR. qRT-PCR was done in triplicate. **D.** PDGF-B protein levels were measured by ELISA in the cell-free BALF. ELISA was done in duplicate. n=3 mice per group. Student's t-test was used.

Figure S7. ***Vhl*** **deletion in myeloid cells increases macrophage Pdgfb, distal muscularization and PH with short-term hypoxia exposure.** *Vhl*$^{(flox/flox)}$ mice also carrying no Cre or *LysM-Cre* were subjected to hypoxia for 7 days. **A**, Pdgfb transcript level of BALF cells was determined by qRT-PCR. **B**, Lung vibratome sections of L.L1.A1.L1 region were stained for SMA and CD31 with boxed regions magnified below. Scale bar, 25 μm. **C, D,** RVSP and Fulton index are shown. n=3-4 mice. Student's t-test was used.

Figure S8. Efficiency of ***Hif*** **deletion in** ***LysM-Cre*** **mice.** *LysM-Cre* mice also carrying floxed alleles for *Hif1a* (**A**) or *Hif2a* (**B**) were exposed to hypoxia (10% FiO_2) for 3 days. BALF was removed, and cellular levels of the indicated transcript were measured by qRT-PCR. n=3 mice for each genotype, qRT-PCR was done in triplicate. Student's t-test was used.

Figure S9. PDGF-B secreted by macrophages from PAH patients induces hPASMC proliferation. Peripheral blood mononuclear cells (PBMCs) were isolated by Ficoll column centrifiguation of fresh whole blood from humans. Monocytes were enriched by allowing cells to adhere to plastic dish for 1 h and discarding floating cells. **A, B,** Using PBMCs from control humans, adherent cells were collected, stained for markers of monocytes (CD14), T-cells (CD3) and B-cells (CD19) and subjected to flow cytometric analysis. n=3 human controls. **C, D**, Monocytes from fresh blood of human controls or PAH patients were differentiated into macrophages, and medium conditioned by these macrophages was collected. In **C**, the levels of PDGF-B protein in the conditioned medium was measured by ELISA and is shown for IPAH and SSc-PAH relative to control. n=3 subjects per PAH diagnostic class and n=6 controls.

ELISA was done in duplicate per subject. One-way ANOVA with Tukey's multiple comparison test was used. In **D**, conditioned medium was incubated with anti-PDGF-B antibody or IgG control for 1 h. hPASMCs were cultured for 24 h with this medium, with BrdU included for the final 10 h of this incubation. Cells were then stained for BrdU and nuclei (propidium iodide [PI]). Scale bar, 20 μm. **E, F,** Cryopreserved PBMCs from PAH patients or human controls were used to generate macrophage conditioned medium, and hPASMCs were cultured for 24 h in this medium. BrdU was included in the last 10 h of this incubation, and cells were then stained for BrdU and nuclei (propidium iodide [PI]). In **E**, the percent of total cells (PI^+ nuclei) expressing BrdU for control humans and PAH patients was normalized to this percentage for controls. In **F,** anti-PDGF-B blocking antibody or control IgG was added to the conditioned medium 1 h prior to incubation with hPASMCs. Results are the ratio of the percent of total (PI^+) cells that are $BrdU^+$ for anti-PDGF-B treatment relative to IgG treatment and normalized to human controls. n=4 PAH patients and n=3 controls (see Table S6). Student's t-test were used. **, ns vs. corresponding IgG controls, <0.01, not significant, respectively. Scale bar, 25 μm.

Figure S10. Nanoparticle administration to mice does not affect lung function. **A**, **B**, Nanoparticles of diameter 200 nm were loaded with DiD and administered orotracheally to normoxic mice. BALF and residual cells were isolated 12 h later and then stained for CD64 and subjected to flow cytometric analysis. **C**, Quantification of experiments represented in **A, B** and Fig. 7A, B showing the percentage of BALF or residual lung (RL) $CD64^+$ cells that contain DiD^+ nanoparticles (diameter 400 or 200 nm as indicated). n=3 mice per treatment. **D-G**, Nanoparticles (diameter 400 nm) loaded with the dye DiD were or were not administered orotracheally to normoxic mice, two times per week for 21 days. Pressure-volume relationships

of the deflation limb (**D**) and lung compliance (**E**) were measured using the flexiVent system, and lung paraffin sections were stained with H&E (**F**). In **G**, vibratome lung sections lacking (#1) or containing (#2) a large caliber airway (aw) were stained for CD64 and the epithelial cell marker aquaporin (AQP)-1. n=3 mice per nanoparticle group. Scale bars, 100 μm (**F**) and 25 μm (**G**).

Figure S11. Orototracheally administered DiD-loaded nanoparticles do not affect the heart or liver. Nanoparticles of diameter 400 nm were loaded with DiD and were or were not administered orotracheally to normoxic mice, two times per week for 19 days. The heart, liver and lung were harvested and cryosectioned. **A,** Heart, liver and lung cryosections stained for CD68 and nuclei (DAPI) are shown. Arrowheads indicate DiD-loaded nanoparticles. **B,** Heart and liver parraffin sections stained with hematoxylin and eosin (H&E) are shown. n=3 mice per nanoparticle group. Scale bars, 25 μm (**A**) and 100 μm (**B**).

Figure S12. siPdgfb attenuates Pdgfb in BALF cells but nanoparticle-mediated Pdgfb knockdown does not alter lung macrophage accumulation. A, BALF cells were isolated from normoxic mice and subjected to siRNA targeting Pdgfb or Scr RNA for 6 h. After 72 h, Pdgfb levels were analyzed by qRT-PCR, which was done in triplicate. **B-D**, Nanoparticles of 400 nm diameter were loaded with siRNA targeting Pdgfb or Scr RNA and then administered orotracheally to mice at the onset of hypoxia and twice per week during the 21 day exposure. In **B, C,** single cell lung suspensions were stained for Ly6G and CD64 and subjected to flow cytometry, and the percent of $CD64^{+}Ly6G^{-}$ macrophages was quantified (**D**). n=3 mice per treatment group. ns, not significant. Student's t-test was used.

Figure S13. siPdgfb-nanoparticles do not affect established pulmonary vascular disease in chronic hypoxia. Mice were exposed to hypoxia for 42 days. Starting on day 21 and twice per week therafter, nanoparticles (400 nm diameter) loaded with siRNA targeting Pdgfb or scrambled (Scr) RNA were administered orotracheally to mice. **A,** Vibratome lung sections containing distal arterioles in the L.L1.A1 area were stained for CD31 and SMA. Boxed regions are shown below as close-ups. Scale bar, 25 µm. **B, C,** RVSP and Fulton index were measured. n=3 mice per treatment group. Student's t-test was used. ns, not significant.

Figure S14. Nanoparticle-mediated knockdown of Pdgfb attenuates RVH in the hypoxia/Sugen 5416 model. Nanoparticles (400 nm diameter) were loaded with siRNA targeting Pdgfb or scrambled (Scr) RNA and then administered to mice at the onset of hypoxia and twice per week during the ensuing 21 days of hypoxia. In addition, immediately prior to hypoxia exposure, mice were injected with a dose of Sugen 5416, and this injection was repeated weekly thereafter. **A,** Lung vibratome sections containing distal arterioles in the L.L1.A1 area were stained for CD31 and SMA. Boxed regions are shown below as close-ups. Scale bar, 25 µm. **B, C,** RVSP and Fulton index were measured. n=3 mice per treatment group. Student's t-test was used. ns, not significant.

Gene	Forward Primer	Reverse Primer
mPdgfb	CATCCGCTCCTTTGATGATCTT	GTGCTCGGGTCATGTTCAAGT
mHif1a	ACCTTCATCGGAAACTCCAAAG	CTGTTAGGCTGGGAAAAGTTAGG
mHif2a	GCCCTACTAAGTGGCCTGTG	GGAGGTTCCAACTGCGATGA
mVhl	CTCAGCCCTACCCGATCTTAC	ACATTGAGGGATGGCACAAAC
mGapdh	TGCACCACCAACTGCTTAGC	GGCATGGACTGTGGTCATGAG
hPDGFB	TCCCGAGGAGCTTTATGAGA	ACTGCACGTTGCGGTTGT
hGAPDH	CTGACTTCAACAGCGACACC	TAGCCAAATTCGTTGTCATACC

Table S1. Primer pair sequences for cDNAs of the indicated genes used in qRT-PCR. For the indicated genes, m and h indicate murine and human, respectively.

Sex	Age	Sample date	PH meds (at sample date)	RHC date	RAP (mmHg)	PAPm (mmHg)	PCWP (mmHg)	CO (L/min)	PVR (Wood)
**IPAH patients**									
F	33	11/15/18	sildenafil, macitentran, selexipag	10/17/16	14	49	8	2.2	18.6
M	53	11/5/18	tadalafil, ambrisentan	6/6/18	12	54	12	4.0	10.5
F	37	12/20/18	tadalafil, ambrisentan	6/20/17	8	48	14	5.1	6.7
F	47	1/7/19	tadalafil, remodulin	7/31/18	15	77	9	2.8	24.3
M	25	10/17/19	sildenafil	8/30/19	11	36	15	3.4	6.1
**SSc-PAH patients**									
F	67	8/1/19	sildenafil, treprostinil	8/9/19	5	38	6	5.2	6.2
F	25	8/1/19	tadalafil, macitentran, treprostinil	8/23/19	9	43	12	6.8	4.6
F	63	10/25/18	tadalafil, ambrisentan	8/30/18	7	37	10	3.8	7.1
F	40	11/1/18	none	10/16/18	9	42	10	4.8	6.7
F	74	11/5/18	none	11/2/18	9	57	11	5.5	8.4
F	80	7/11/19	none	7/7/19	3	21	8	3.4	3.8

Table S2. Human PAH patients - PH meds and right heart catheterization (RHC) data.
Age is in years. RAP, right atrial pressure; PAPm, mean pulmonary artery pressure; PCWP, pulmonry capillary wedge pressure; CO, cardiac output; PVR, pulmonary vascular resistance.

Sex	Age (years)	Sample date	WBC date	WBC (10^3 cells/µl)	Neuts (%)	Lymphs (%)	Monos (%)	Eos (%)	Basos (%)
**IPAH patients**									
F	33	11/15/18	10/19/18	7.0	63.6	27.4	7.6	0.6	0.5
M	53	11/5/18	10/22/18	7.9	73.1	17.8	7.9	2.3	0.5
F	37	12/20/18	8/17/17	6.6	63.9	26.7	4.7	3.9	0.5
F	47	1/7/19	3/9/19	13.7	89.3	5.3	3.6	0.4	0.2
M	25	10/17/19	8/30/19	6.0	57.9	28.3	11.9	1.2	0.5
**SSc-PAH patients**									
F	67	8/1/19	8/1/19	7.0	78.2	6.3	9.6	4.7	0.9
F	25	8/1/19	8/1/19	1.7	65.3	28.3	5.8	0.0	0.0
F	63	10/25/18	10/9/18	4.9	75.3	13.3	8.6	2.0	0.4
F	40	11/1/18	10/16/18	4.4	57.7	30.0	8.4	2.5	0.9
F	74	11/5/18	10/5/18	5.0	50.0	29.2	11.5	8.3	1.0
F	80	7/11/19	7/11/19	5.7	59.2	31.6	7.5	0.9	0.4

Table S3. Human patients - white blood cell (WBC) count and differential

Sample date is the date fresh blood was drawn for PBMC isolation.

Controls

Sex	Age (years)	Relative Pdgfb mRNA level
M	30	0.654
F	30	0.732
F	41	0.869
M	55	0.934
F	72	0.975
M	32	1.131
F	36	1.177
F	37	1.203
M	50	1.325
	Average:	1.000
	Standard deviation:	0.226

Patients

IPAH

Sex	Age (years)	Relative Pdgfb mRNA level
M	53	2.223
F	33	5.030
F	47	5.485
F	37	6.466
M	25	6.529
	Average:	5.147
	Standard deviation:	1.755

SSc

Sex	Age (years)	Relative Pdgfb mRNA level
F	25	5.923
F	74	6.885
F	67	9.172
F	63	14.661
F	80	16.880
	Average:	10.704
	Standard deviation:	4.837

Table S4. Human subjects (fresh blood samples) for Pdgfb mRNA assay.
Values are normalized to controls.

Controls

Sex	Age (years)	Relative BrdU+ cells
F	30	0.891
F	72	0.956
F	41	0.956
M	55	0.974
F	36	1.039
F	37	1.185
	Average:	1.000
	Standard deviation:	0.102

Patients

IPAH

M	53	4.296
F	33	4.562
F	37	4.907
	Average:	4.588
	Standard deviation:	0.306

SSc

F	40	5.054
F	74	7.009
F	25	8.902
	Average:	6.988
	Standard deviation:	1.924

Table S5. Human subjects (fresh blood samples) for proliferation assay.
Values are normalized to controls.

Controls

Diagnosis	Sex	Age (years)	Relative BrdU+ cells
Control	F	23	0.684
Control	F	72	1.104
Control	F	42	1.212
		Average:	1.000
		Standard deviation:	0.279

Patients

PAH

Diagnosis	Sex	Age (years)	Relative BrdU+ cells
IPAH	F	54	1.438
SSc-PAH	F	45	1.482
IPAH	F	51	1.518
IPAH	F	24	1.785
		Average:	1.556
		Standard deviation:	0.156

Table S6. Human subjects (cryopreserved samples) for proliferation assay.
Values are normalized to controls.

Controls

		Relative migration	
**Sex**	**Age (years)**	**IgG**	**Anti-PDGF-B**
F	36	0.950	1.131
F	72	1.432	1.387
F	41	0.618	0.995
	Average:	1.000	1.171
	Standard deviation:	0.576	0.199

Patients

IPAH

F	37	2.397	1.658
M	53	3.317	1.508
F	33	2.171	1.643
F	47	3.950	1.176
	Average:	2.959	1.496
	Standard deviation:	0.826	0.224

SSc

F	25	3.874	2.276
F	74	5.322	2.472
F	63	3.709	3.181
F	40	3.754	1.930
	Average:	4.165	2.465
	Standard deviation:	0.775	0.527

Table S7. Human subjects (fresh blood samples) for migration assay.
Values are normalized to IgG controls.
